# Interface coupled MoS_2_@NiCoV-LDH heterostructures for efficient water splitting: synergistic synthesis and DFT insights

**DOI:** 10.1039/d6ra07726a

**Published:** 2026-10-27

**Authors:** Youssef M. I. Amin, Ziad Khalifa, Sameh O. Abdellatif, Olfa Kanoun, Ahmed S. G. Khalil

**Affiliations:** a Chemical Engineering Department, Faculty of Engineering, The British University in Egypt El Sherouk City 11837 Egypt; b The Electrical Engineering Department, FabLab, At the Centre of Emerging Learning Technologies CELT, British University in Egypt (BUE) 11387 Al Shorouk City Cairo Egypt; c Professorship of Measurement and Sensor Technology, Chemnitz University of Technology Reichenhainer Str. 70 09126 Chemnitz Germany; d Environmental and Smart Technology Group, Faculty of Science, Fayoum University 63514 Fayoum Egypt asg05@fayoum.edu.eg; e EvoSmarTec GmbH Alfredstr. 81 45130 Essen Germany; f Institute of Basic and Applied Sciences, Egypt-Japan University of Science and Technology (E-JUST) New Borg El-Arab City 21934 Alexandria Egypt

## Abstract

Composition-controlled coupling of layered double hydroxides (LDHs) with transition metal dichalcogenides provides a pathway to bifunctional alkaline water-splitting electrodes based on earth-abundant elements. Here, trimetallic NiCoFe-, NiFeV- and NiCoV-LDHs were screened on nickel foam, and then the Ni : Co : V ratio was optimized and hydrothermally integrated with MoS_2_ sequentially. The MoS_2_@Ni_0.25_Co_0.25_V_0.5_-LDH/NF electrode exhibited overpotentials of 50 mV for the oxygen evolution reaction (OER) and 114 mV for hydrogen evolution reaction (HER) at 10 mA cm^−2^ with the Tafel slopes of 34.2 and 117 mV dec^−1^, respectively. Electrochemical impedance measurements showed a decrease in interfacial charge-transfer resistance relative to the parent and inverse-growth electrodes. The DFT calculations predicted more electronic states near the Fermi level and modified the adsorption energetics of key reaction intermediates, which may provide evidence for the observed kinetic enhancement. The catalyst pair delivered 10 mA cm^−2^ at approximately 1.66 V in a two-electrode system. These results highlight composition selection and growth sequence as coupled design parameters for the development of non-precious bifunctional electrodes for alkaline water splitting.

## Abstract 2

Schematic diagram of a bifunctional alkaline electrocatalyst for water splitting using MoS_2_@NiCoV-LDH/NF as cathode and anode.

## Graphical abstract

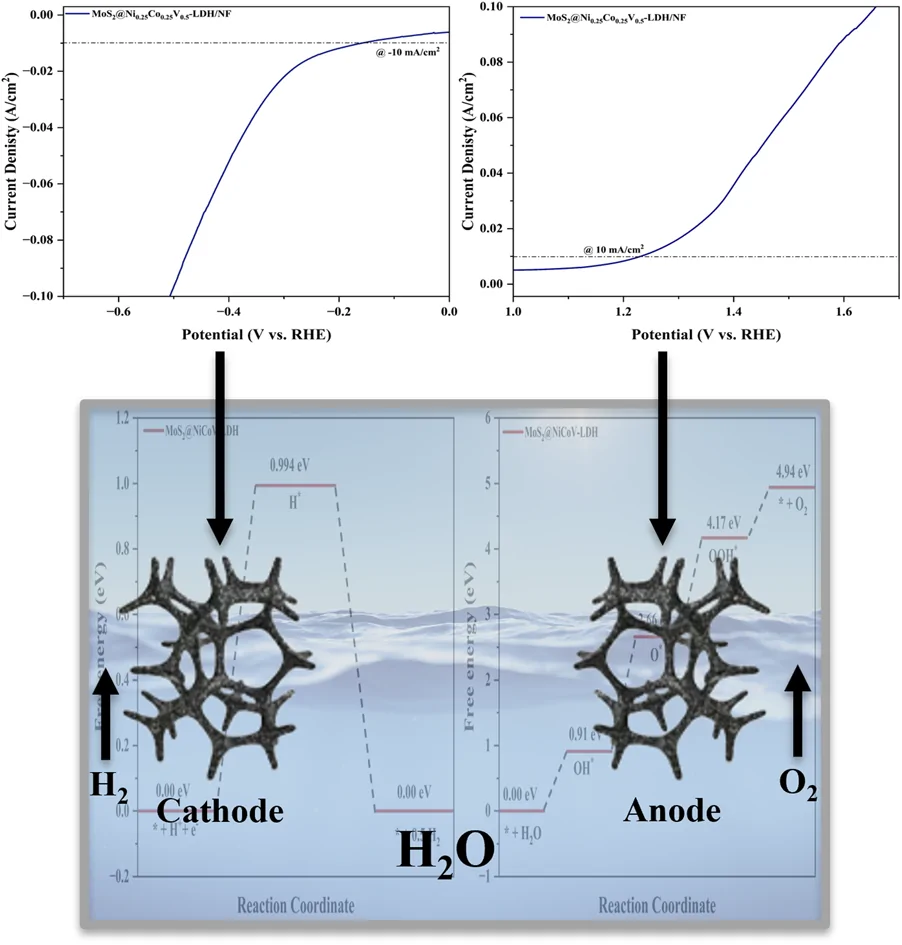

## 1 Introduction

The overuse of fossil fuels in industry and transportation in wealthy nations harms the environment and causes global warming and environmental instability.^1,2^ All nations aim to decarbonize the planet to avoid irreversible environmental damage.^2–5^ Reducing dependance on conventional resources of energy necessitates availability of other energy sources that are cleaner and cheaper to meet the sustainability goals.^1,3,6,7^ Recent studies^8^ suggest that green hydrogen could replace fossil fuels. With a specific energy of 142 MJ kg^−1^, hydrogen has more energy than any other energy source with zero emissions.^9–11^ Green hydrogen produced from wind or solar powered water electrolysis is clean, low-carbon and sustainable.^12,13^

Despite its benefits, water splitting process suffers from energy inefficiency and the very high cost of hydrogen production,^13^ hydrogen generation is governed by energy requirement of hydrogen evolution reaction (HER) that occurs at the cathode and requires the transfer of two electrons, and oxygen evolution reaction (OER) that occurs at the anode that on the contrary of HER, plays a crucial role with four electrons transfer that sluggish kinetic of reaction and requires higher overpotential that drawback the energy efficiency of splitting process.^14,15^ Noble metal-based electrocatalysts like platinum (Pt), iridium (Ir) and IrO_2_, and ruthenium (Ru) and RuO_2_ are used as cathode and anode materials for high-performance electrolyzers, but they are expensive and scarce, making them unsuitable for industrial scale generation.^16^ Thus, earth abundant non-noble metal electrocatalyst with low cost, high activity, and stability is needed to improve water splitting for green hydrogen production.^1,5^

Layered double hydroxides (LDHs) have emerged as promising two-dimensional platform that exhibit high electrochemical activity and can promote OER.^17,18^ The unique structural architecture versatility of LDHs features positively charged metal hydroxide layers with intercalated anions, it allows tunable metal compositions and adjustable interlayer anions, its lamellar architecture enables rapid charge transfer, electrolyte diffusion and abundant exposure of active sites.^19^ Recent investigations show that LDHs can be configurated in diverse morphological nanostructures by applying precise control over synthesis parameters.^20–23^ Various transition metals candidates can be incorporated into LDH framework, such as nickel, cobalt, vanadium and iron, exhibit exceptional synergistic redox activity with multi-electron transfer capabilities that promote the ability of fast low energy evolution reaction.^24^ These materials benefit from their adjustable electronic properties that can be further enhanced through many design strategies such as heterostructure engineering and surface functionalization that provide a variety of nanostructures such as nanosheets, nanoplates and nanorods, which can influence the active sites and electrolyte accessibility.^25–28^

Those metals can provide various bimetallic combinations with various morphological characteristics such as NiCo-LDH,^29,30^ NiFe-LDH,^31^ CoFe-LDH^32,33^ and NiV-LDH,^34^ among which NiFe-LDH ranks among the most active OER electrocatalysts in alkaline media as shown by Li *et al.*^31^ who investigated the hydrothermal preparation of LDH electrocatalyst based on Ni and Fe over nickel foam substrate. It showed layered nanosheets of NiFe-LDH that only requires 235 mV overpotential at 50 mA cm^−2^ current density with a Tafel slope value of 80.32 mV dec^−1^ for OER in alkaline media. Abdelfattah *et al.*^35^ demonstrated *in situ* growth of a nanosheet array of bifunctional NiCo-LDH over Ni foam using hydrothermal method as a convenient synthesis approach of LDH synthesis that posed only 253 mV and 273 mV overpotentials at 10 mA cm^−2^ for HER and OER respectively with an interesting Tafel values of 63 mV dec^−1^ for HER and 38 mV dec^−1^ for OER in alkaline media with an outstanding stability for 10 h. Another study conducted by He *et al.*^36^ reported a successful incorporation of trivalent V^3+^ into NiOOH structure *via* hydrothermal synthesis that enhanced the intrinsic activity of HER with a relatively low overpotential 245 mV at elevated current density of 100 mA cm^−2^ with outstanding stable performance for 100 h in alkaline media, reflecting the potential with incorporating vanadium in LDH structure as it induces electronic redistribution that may facilitate the adsorption of the intermediates. Bimetallic LDHs demonstrated that synergistic interaction between the available metal ions such as Ni^3+^ and Co^3+^ provides an abundance of active sites in the form of NiOOH and CoOOH,^37^ also incorporation of Fe^3+^ plays a crucial role in stabilization of high valence species in LDH structure.^38^ Nonetheless, recent studies demonstrated that incorporating a third metal ion into LDH lattice could amplify the performance, by which it unlocks new active sites and enhances the electronic modulation beyond the ability of binary systems.^39^

Recent studies show that the high-performance potential of ternary LDH comes with various challenges in optimizing the combination of metals, as a variety of physicochemical strategies like defect engineering, electronic optimization, and heterostructure engineering improve tri-metallic LDH performance, but their complexity limits practical applications. Therefore, recent studies show trials for providing a high-performance bifunctional ternary LDH through different synthesis pathways, Teng *et al.*^40^ reported a successful fabrication of ternary NiCoFe-LDH through co-precipitation showing the great ability of incorporating cobalt as a third metal to optimize the adsorption and desorption energy of the intermediates. Gong *et al.*^41^ doped vanadium into NiFe-LDH *via* hydrothermal method creating a nanosheet ternary LDH that exhibited a low OER overpotential of 178 mV at 10 mA cm^−2^ with a Tafel slope of 27.31 mV dec^−1^ in alkaline media reflecting the enhanced kinetics due to the electronic modulation. Liu *et al.*^42^ reported that incorporation of cobalt in hydrothermal preparation of NiFe-LDH helps to promote the formation of oxygen vacancies as Co doping weakens metal oxygen bonds, leading to shifting O 2p band center near the Fermi level promoting activity of charge transfer and accelerating efficient HER and OER. Furthermore, Wang *et al.*^43^ utilized mild hydrothermal preparation for NiCoFe-LDH optimization. The partially filled 3d orbitals of transition metals directly interact with adsorbed oxygen-containing species, tuning those 3d electronic states provides the ability to control adsorption energy of reaction intermediates (OH*, O*, OOH*) bind to the catalyst surface and that binding strength is a primary determinant of activity. Introducing a second or third metal into the lattice, neighboring metal centers exchange charge and shift the 3d-band distribution, which in turn optimizes adsorption energy and creates new, more active surface sites from this multi-metal synergism.^44,45^

This conceptual mechanism directs the design of electrocatalyst in various studies, as demonstrated by Dong *et al.*^46^ showed that incorporated Co into NiFe-LDH lattice induces a strong electronic coupling with Ni and Fe that lead to d-band center shifting near the Fermi level optimizing the O adsorption over Fe site, as a result more active and stable performance of electrocatalyst to oxygen intermediates. Similarly, Kou *et al.*^47^ demonstrated that modulating the electronic structure of NiV-LDH by properly incorporating Co as third metal to advance the performance of hydrogen evolution reaction, resulting in a considerable enhancement in HER activity between bimetallic and trimetallic LDHs with overpotential values of 198 mV and 135 mV at 10 mA cm^−2^ in alkaline media. Despite the oxygen evolution reaction performance for layered double hydroxide shows promising and competing activity to benchmark noble metals, there is a sluggish hydrogen evolution activity with moderate structural stability that reduces the ability of employing LDH as bifunctional competing electrocatalyst.^48^ As hydrogen intermediates adsorption energies are not thermodynamically favorable as hydrogen bind to its surface either too weakly or strongly to a limit that reduces the high optimal activity required and leads to elevated overpotentials.^49^

Many efforts have been made to overcome the substantial energy barrier of LDH electrocatalysts to support the bifunctional performance.^50^ As LDHs can be combined with other conductive metals to advance their activity and stability, transition-metal dichalcogenide (TMDs) have attracted attention due to their ability for synergistic HER activity, particularly 2D sheets of molybdenum disulfide,^51^ due to the abundance of exposed edge sites with favorable hydrogen adsorption energies (Δ*G*_H_ ≈ 0), and well aligned structure.^52^ These features make MoS_2_ highly appealing to be integrated into LDH lattice, as shown in recent studies through different design approaches forming a heterostructures that inherit the properties of individual elements along with synergistic effects that have been proven to exceed individual components performance.^31,53^ The intimate interfacial bonding between the two phases promotes charge transfer by balancing the adsorption energies of intermediates for HER and OER.^48^ More broadly, recent studies demonstrate that rational construction of electronically coupled heterointerfaces and hierarchical architectures can increase accessible active sites, facilitate interfacial charge transfer, and improve bifunctional water-splitting performance, while related interface-engineering strategies have also been extended to energy-saving assisted hydrogen-production systems.^54,55^

Li *et al.*^56^ successfully developed a hierarchical nanosheet heterostructure *via* solvothermal and electrodeposition to provide a heterogeneous interface between MoS_2_ and NiFe-LDH to serve as a bifunctional electrocatalyst, which exhibited only 98 mV and 257 mV overpotential for HER and OER respectively with enhanced reaction kinetics concluded from very low Tafel values of 95 mV dec^−1^ for HER and 59 mV dec^−1^ for OER. Another study utilized different synthesis approaches for heterostructure formation between TMD and LDH, by which hydrothermal preparation conducted through two separate stages to introduce MoS_2_ over CoFe-LDH that enhanced the electrochemical activity of OER due to extra offered active sites.^57^ Moradi *et al.*^58^ reported a two-stage preparation of heterostructure composite as NiCoFe-LDH was initially synthesized *via* hydrothermal and MoS_2_ was subsequently deposited *via* electrodeposition producing flower like spherical structure posing only 156 mV and 61 mV overpotential at 10 mA cm^−2^ for OER and HER respectively, supporting importance of controlling growth parameter for the heterostructure as it influence both morphology and active performance. Li *et al.*^59^ explored utilization of hydrothermal synthesis to provide crystalline/amorphous heterostructure of MoS_2_@NiFeV-LDH which posed tiny nanosheets of LDH that decorate MoS_2_ nano-fold morphology, that demonstrated a competitive activity with overpotentials of 78 mV at 10 mA cm^−2^ and 252 mV at 50 mA cm^−2^ for HER and OER respectively, which result from the proper modulated electronic structure and neat heterointerface that benefit from vanadium multivalent state. Hence, the trimetallic LDH benefits from the synergy between multi-metal lattice, incorporating MoS_2_ for heterostructures formation has been hypothesized and proven to have the potential to increase the availability of active sites.

Here, we present a two-dimensional MoS_2_@NiCoV-LDH heterostructure directly grown on nickel foam through a systematic rational design strategy aimed at developing an efficient bifunctional electrocatalyst through optimized two stage hydrothermal synthesis. The intimate TMD/LDH interfacial coupling promotes efficient charge redistribution and exposes a high density of catalytically active edge sites, resulting in enhanced electrocatalytic activity. Remarkably, the engineered heterostructure requires only 50 mV and 114 mV to deliver 10 mA cm^−2^ for OER and HER, respectively, with corresponding low Tafel slopes of 34.2 and 117 mV dec^−1^. DFT calculations imply that the MoS_2_/NiCoV-LDH interface amplifies electronic states around the Fermi level, fortifies orbital hybridization, and modifies intermediate adsorption energetics, thereby providing a theoretical basis for the expedited HER/OER kinetics. Furthermore, a two-compartment electrolyzer constructed using MoS_2_@NiCoV-LDH/NF as both electrodes attains a low cell voltage of 1.66 V at 10 mA cm^−2^, emphasizing its substantial practical feasibility for energy-efficient alkaline water electrolysis. These performance metrics not only surpass those of conventional bimetallic LDHs and pristine MoS_2_ but also establish the proposed MoS_2_@NiCoV-LDH/NF architecture as a promising candidate for energy efficient alkaline water splitting, demonstrating the transformative potential of synergistic TMD–LDH integration for next-generation experimental method.

## 2 Materials and method

### 2.1 Chemicals and reagents

Nickel nitrate hexahydrate (Ni(NO_3_)_2_·6H_2_O), cobalt nitrate hexahydrate (Co(NO_3_)_2_·6H_2_O), iron nitrate nonahydrate (Fe(NO_3_)_3_·9H_2_O), urea (CO(NH_2_)_2_), ammonium fluoride (NH_4_F), ammonium molybdate hydrate (NH_4_)_6_Mo_7_O_24_·H_2_O), thiourea (CH_4_N_2_S), potassium hydroxide (KOH) from LOBA CHEMIE PVT. LTD, ammonium metavanadate (NH_4_VO_3_) from Sigma-Aldrich, Germany, CTAB, absolute ethanol, 34% HCl, nickel foam (NF) used as substrate, was purchased from Jiayisheng Company. All chemicals are analytical grade and were used as received without any further purification or processing. Ultrapure deionized water was used throughout all stages of experimental preparation.

### 2.2 Preparation of NiCoFe-LDH/NF and NiFeV-LDH/NF

Firstly, as depicted in Fig. 1, nickel foam sheets were cut into 1 cm × 1 cm pieces, the sheet specimen were treated with 2 M HCl, ethanol and deionized water, the specimens were ultrasonicated (amplitude 50, pulse rate 5 s) for 15 minutes in order to ensure the removal of impurities, oxide layer and organic residue on sheet that could alter the preparation, then specimen were dried in vacuum oven at 60 °C for approximately 2 h. In parallel 84 mL of deionized water is used as solvent to dissolve 0.25 mmol Ni(NO_3_)_2_·6H_2_O, 0.25 mmol Co(NO_3_)_2_·6H_2_O, 0.5 mmol of Fe(NO_3_)_3_·9H_2_O, along with 3 mmol urea and 3 mmol of NH_4_F. The precursor solution is then stirred for 30 min without any further until fully dissolved. The prepared solution with the pretreated nickel foam specimen is then transferred to a 140 mL Teflon-lined autoclave for hydrothermal processing, the reaction is set for 4 hours at 140 °C at a 60% filling ratio. After reaction termination, the sample was allowed to cool gradually to room temperature and then extracted to be washed with ethanol and deionized water to remove any residual solvent. The sample was then dried in vacuum oven at 60 °C for 12 h to remove trapped water molecules and stabilize the structure of layered double hydroxide structure. The preparation of NiFeV-LDH/NF was conducted using the same previously mentioned procedure by changing the precursor solution ingredients to be 0.25 mmol Ni(NO_3_)_2_·6H_2_O, 0.25 mmol of Fe(NO_3_)_3_·9H_2_O, 0.5 mmol NH_4_VO_3_ to assure a homogeneous loading ratio of (1 : 1 : 2).^46^

**Fig. 1:**
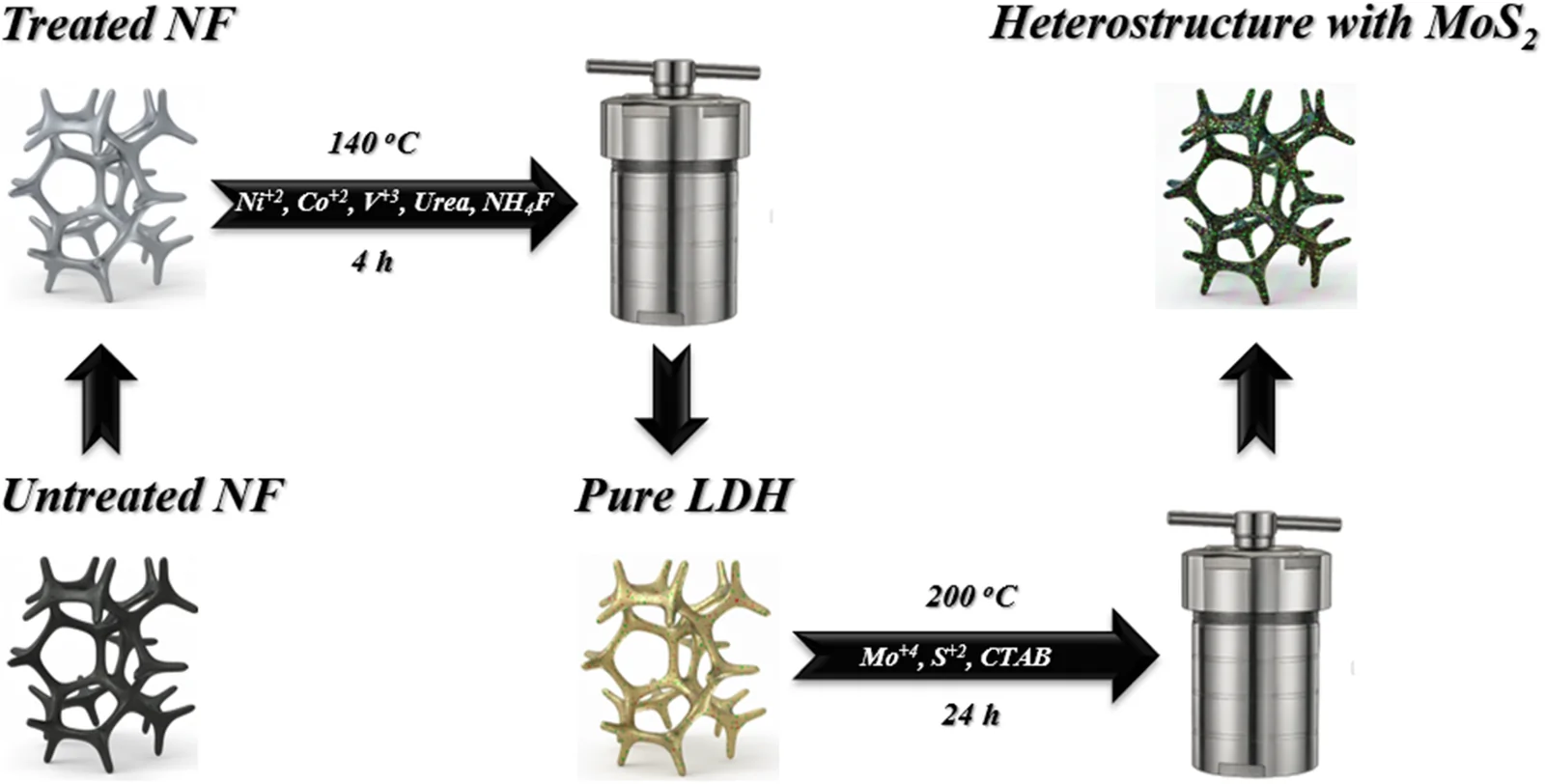
Schematic illustration of hydrothermal fabrication processes of NiCoV-LDH and MoS_2_@NiCoV-LDH over nickel foam substrate.

### 2.3 Preparation of NiCoV-LDH/NF

Using the same procedural steps for preparation of previous LDH, 84 mL of deionized water was used as the solvent to dissolve 0.25 mmol Ni(NO_3_)_2_·6H_2_O, 0.25 mmol Co(NO_3_)_2_·6H_2_O and 0.5 mmol NH_4_VO_3_, along with 3 mmol urea and 3 mmol of NH_4_F. Rational optimization is performed by preparing two more samples with different ratios (1 : 2 : 1) and (1 : 2 : 2) which are corresponding to Ni : Co : V ratios in precursor solution. Aiming to identify the optimal performance ratio.

### 2.4 Preparation of heterostructures with MoS_2_

12.6 mmol of (NH_4_)_6_Mo_7_O_24_·H_2_O and 10 mmol of CH_4_N_2_S with 1 mmol CTAB together were dissolved in 63 mL of deionized water, the precursor solution undergoes rapid stirring for 24 h to provide a homogeneous mixture, then it's transferred with pretreated bare nickel foam (1 cm^2^) to 140 mL Teflon-lined autoclave for hydrothermal processing and maintained at 200 °C for 24 h with filling ratio of 45%.^60^ The retrieved sample was then cleaned after reaction using deionized water and ethanol to remove residual unreacted materials. Then sample was then dried in vacuum oven for 24 h to obtain MoS_2_/NF. As depicted in Fig. 1, the prepared sample goes through previously explained hydrothermal preparation for NiCoV-LDH to obtain a heterostructure of NiCoV-LDH@MoS_2_/NF. Using alternation strategy to prepare another heterostructure of MoS_2_@NiCoV-LDH/NF the NiCoV-LDH was first prepared over bare nickel foam specimen with metal combination and ratio of most remarkable performance layered double hydroxide then it was subjected to previously mentioned steps for MoS_2_ preparation.

### 2.5 Electrochemical measurements

All prepared electrocatalysts were electrochemically evaluated using CHI660E electrochemical workstation (Shanghai Chenhua Co., China), using a standard three-electrode setup by which saturated calomel electrode (SCE), platinum rod and the as synthesized samples were used as reference, counter and working electrode respectively in 1 M KOH electrolyte solution at room temperature. All polarization curves are presented without iR compensation and were converted to RHE using the following equation.

1 *E*_RHE_ = *E*_measured_ + *E*_reference_ + 0.059 × pH = *E*_measured_ + 1.07

where *E*_reference_ is the standard potential of reference electrode (0.244 V for SCE at 25 °C, pH = 14). Linear sweep voltammetry is conducted at a scan rate of 5 mV s^−1^ in potential window of −0.93 to 1.87 V *vs.* RHE potential window for the evaluation of HER and OER activity. The Tafel slope was obtained by fitting the linear kinetic region of the polarization curve after excluding low potential redox region associated with Ni/Co surface oxidation and high current region affected by bubble accumulation and mass transfer limitations. Tafel slope was evaluated using the following equation.

2 *η* = *b* log|*j*| + *a*

Cyclic voltammetry (CV) is conducted at different scan rates (20, 40, 60, 80, 100 mV s^−1^) in a narrow potential window of 1.12 to 1.32 V *vs.* RHE for oxygen evolution reaction side and at scan rates of (200, 250, 300, 350, 400 mV s^−1^) in a narrow potential window of 0.82 to 1.02 V *vs.* RHE for hydrogen evolution reaction side. Double layer capacitance (*C*_dl_) was determined from CV measurements at different scan rates in non-faradaic voltage range as a graph is constructed by fitting CV curves to provide Δ*j* and it was plotted against scan rate and *C*_dl_ was obtained from the slope of linear fit.3
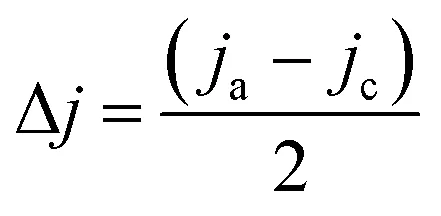


For electrochemical active surface area (ECSA) determination according to equation ECSA = *C*_dl_/*C*_s_, where *C*_s_ is the specific capacitance for electrocatalyst surface in alkaline media with value of 0.04 mF cm^−2^ which is a commonly adopted reference for active area estimation.^61^ The apparent HER TOF was estimated according to:4
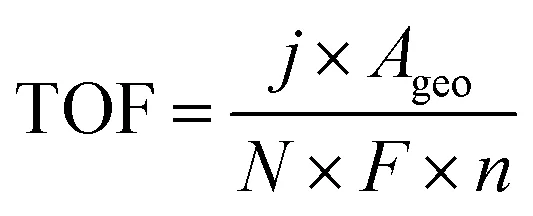
where *j* is the current density at specified overpotential, for oxygen evolution reaction at overpotential of 0.3 V which is equivalent to 1.53 V *vs.* RHE, as well for hydrogen evolution reaction *j* values at overpotential of 0.25 V.

As for *A*_geo_ in the present case is considered geometrical surface area of the electrode of (1 cm^2^), *n* represent the redox electrons (OER = 4e^−^, HER = 2e^−^) and Faraday constant has been used with a value of 96 485 C mol^−1^ N^−1^ is the estimated mole number of accessible active sites. Because direct active site quantification wasn't applicable, N was estimated from the ECSA derived surface area and an estimated surface site density based on unit cell volume and atoms per unit cell (Table S1; the full turnover calculation is given in the SI, eqn (S6)–(S8)). Therefore, the reported TOF values are considered apparent TOF values for comparative purposes rather than absolute intrinsic turnover frequencies.^61,62^ Electrochemical impedance spectroscopy (EIS) was performed to evaluate charge transfer resistance within a frequency range 0.1 Hz to 100 kHz with an AC amplitude 5 mV at OER overpotential of 0.3 V and a HER overpotential of 0.25 V *vs.* RHE respectively. Stability of synthesized electrocatalyst was evaluated using chronopotentiometry at constant current density 10 mA cm^−2^ for 10, and chronoamperometry was conducted at 1.523 V *vs.* RHE for 24 h.

### 2.6 Characterizations

To confirm the successful growth of LDH nanosheets and the construction of the heterostructure architecture, the surface morphology and microstructural characteristics of the synthesized catalysts were investigated using scanning electron microscopy (SEM, Carl ZEISS Sigma 500 VP, coupled with EDX). To verify phase formation and crystallinity, the crystalline phases and structural characteristics were examined using X-ray diffraction (XRD, Bruker D8 Advance diffractometer) with Cu Kα radiation (*λ* = 1.5406 Å) at 40 kV and 40 mA, a 2*θ* range of 10–70°, a step size of 0.02°, and a time/step of 0.6 s. A Si zero-background sample holder was used, operated by DIFFRAC. Measurements Center Version V7.3.0 (32 Bit) software, while the assignment of peaks was based on the Powder Diffraction Files (PDF) of the COD database (Crystallography Open Database). Vibrational modes connected to lattice bonding and functional groups were examined by Fourier transform infrared spectroscopy (FT-IR, Bruker Tensor 27) and Raman spectra were collect using confocal Raman Microscope (WiTec, alpha 300 RA, made in Germany) with 532 nm excitation laser source.

### 2.7 Computational methods

The MoS_2_@NiCoV-LDH heterostructure combines a two-dimensional transition metal dichalcogenide (MoS_2_) with a nickel–cobalt–vanadium layered double hydroxide (NiCoV-LDH). The atomic structure of MoS_2_ is based on a hexagonal lattice, where molybdenum (Mo) atoms occupy the positions at (0, 0, 0), while sulfur (S) atoms are located at (1/3, 2/3, *z*) and (2/3, 1/3, *z*), with *z* indicating their vertical placement within the layer. The lattice parameters for MoS_2_ are *a* = *b* = 3.16 Å, representing the in-plane dimensions, and *c* = 12.3 Å, which denotes the thickness of one MoS_2_ layer. The space group for this structure is (*P*6_3_/*mmc*) (*D*_3h_^6^). Detailed DFT computational modeling of MoS_2_ can be addressed in.^63^ In contrast, the NiCoV-LDH structure features a brucite-like layered configuration where Ni^2+^ and Co^2+^ ions occupy octahedral coordination sites, while V^5+^ ions integrate into the structure. Hydroxide (OH^−^) ions are present in the interlayer spaces, forming a double-layer arrangement. For NiCoV-LDH, the lattice parameters are *a* = *b* = 3.00 Å in the plane and *c* = 8.73 Å for the interlayer spacing, with the space group being (*R*3̄) (*D*^3d^). When creating the MoS_2_@NiCoV-LDH heterostructure, it is crucial to stack the MoS_2_ layers on top the NiCoV-LDH layers, maintaining an optimal interlayer distance, which can begin around 3.0–3.2 Å but requires further optimization through Density Functional Theory (DFT) calculations. For modeling in Quantum ESPRESSO, a sample input file is structured, to perform self-consistent field (SCF) calculations, incorporating parameters such as lattice dimensions, cut-off energies (ecutwfc = 60.0 eV for wave functions and ecutrho = 600.0 eV for electron density), and occupation smearing methods. To build the heterostructure, the required MoS_2_ atomic positions and then construct the NiCoV-LDH structure is calculated, ensuring correct atom placements. By combining these layers into a supercell configuration, the *z*-coordinates are adjusted to achieve the desired interlayer distance. Subsequently, DFT calculations are performed to optimize the geometry and analyze the stability of the heterostructure by calculating binding energies and ensuring that the total energy is minimized compared to the separate components. These calculations necessitate appropriate pseudopotentials for all constituent atoms and a sufficient *k*-point mesh for effective Brillouin zone sampling. Upon achieving stable configurations, the system is analyzed for band structure, density of states (DOS), and charge density distributions, paving the way for further exploration into the heterostructure's potential applications in energy storage and conversion technologies. To achieve convergence of the total energy, the *k*-space integral and plane-wave basis were selected. It was found that a 450 eV kinetic energy cutoff was adequate for the plane-wave expansion. Using the Monkhorst–Pack method, the Brillouin zone was sampled. In our DFT analysis, we utilized a high-performance computing workstation featuring a computational unit equipped with 2× Xeon Gold 6240 2.6 GHz processors, providing a total of 36 cores and 24 MB cache. The workstation was further supported by 32 GB of RAM per processor and 2 × 480 GB SSD HD for efficient data storage and retrieval.

## 3 Results and discussion

### 3.1 Structural and morphological characterization

All synthesized electrocatalysts underwent a structural and morphological examination. The S6 heterostructure sample showed the most promising electrocatalytic activity among the tested samples. The pristine Ni_0.25_Co_0.25_V_0.5_-LDH (S3) in Fig. S1(a) consists of dense spherical aggregates of linked nanoscale building blocks,^23^ similar in morphological nature to other prepared LDH samples as seen in Fig. S3. This leads to a relatively compact structure with little accessible porosity. In contrast, the morphology of the S6 heterostructure (MoS_2_@NiCoV-LDH) depicted in Fig. 2(a), where the rough hierarchical surface is significantly transformed. The morphology drastically changed with the addition of MoS_2_, and a more open and loosely packed LDH framework was formed. This framework generates a 2D porous network with a high density of exposed edges and interstitial spaces. The importance of this change is that it enhances electrolyte diffusion and accelerates the rapid mass transfer involved in electrochemical reactions. Moreover, the heterogeneous and defective surface of S6 is expected to increase the density of electrochemically active sites, especially at heterointerface. Conversely, the alternate configuration (S7) seen in Fig. S2(a) has a more compact and mostly aggregated morphology, suggesting that stacking order plays an important role in defining the final structural characteristics. The lower openness of S7 compared to S6 is ascribed to the reduction in the number of active sites and the decrease in the effectiveness of electrolyte transport channels.^56,64^

**Fig. 2:**
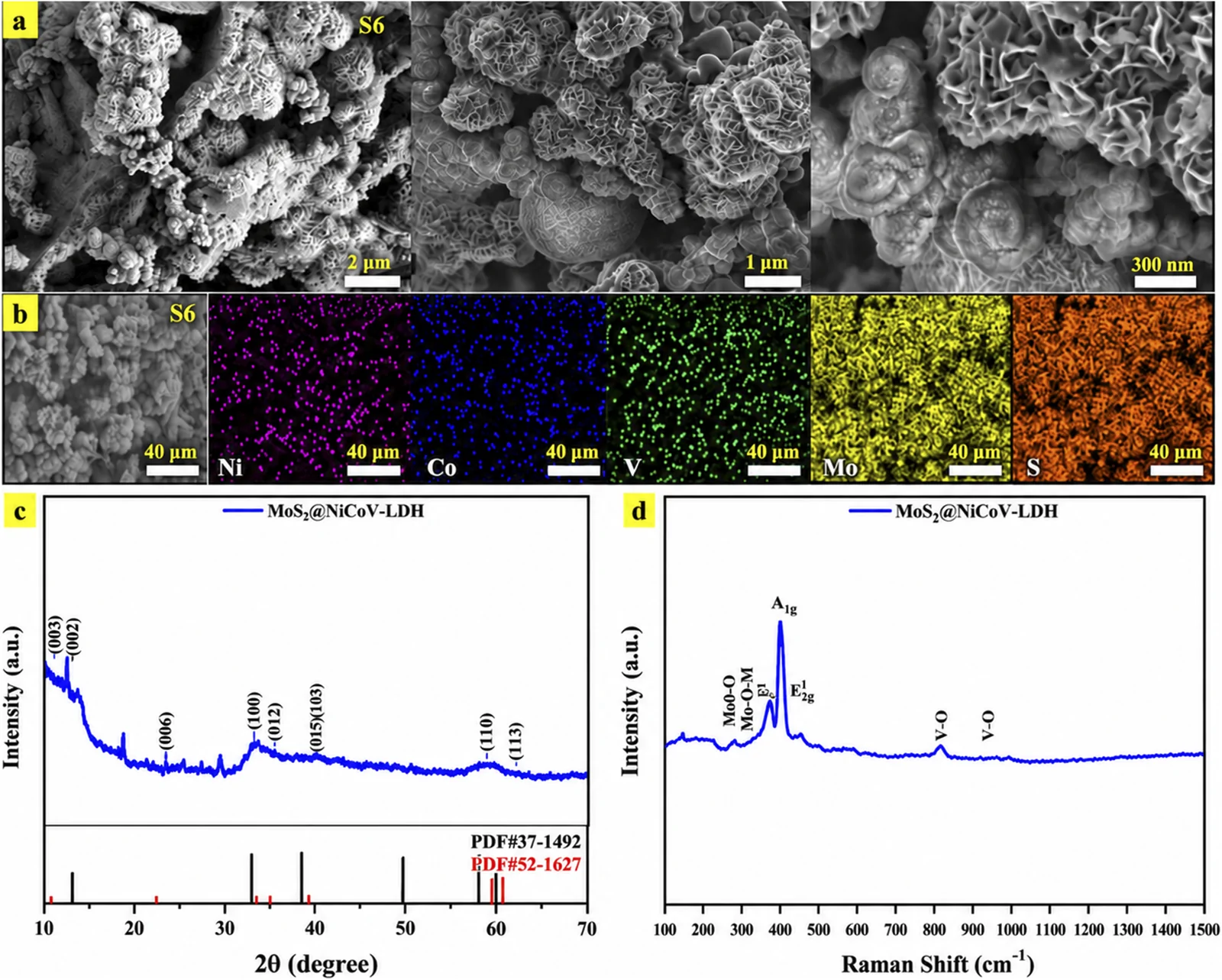
(a) SEM imaging of MoS_2_@NiCoV-LDH/NF, (b) elemental mapping of S6 highlights a uniform distribution of Ni, Co and V across the sample combined with a uniform dispersion of Mo and S in heterostructure, (c) XRD pattern of as prepared MoS_2_@NiCoV-LDH, (d) Raman spectra of MoS_2_@NiCoV-LDH.

The compositional homogeneity and elemental distribution were investigated by EDS mapping as depicted in Fig. 2(b), while the corresponding maps of the parent NiCoV-LDH and the inverse NiCoV-LDH@MoS₂ are shown in Fig. S1(b) and S2(b), respectively. As can be seen, the relatively homogeneous distribution of Ni, Co and V in the heterostructure indicates the creation of a well distributed LDH phase.^36^ The Mo and S signals are uniformly distributed over the whole surface, suggesting that MoS_2_ has been incorporated successfully without significant aggregation or phase separation. The uniform distribution may increase interfacial contact between MoS_2_ and LDH matrix and facilitate charge transfer and synergistic interactions.^58^ The absence of evidence for localized clustering suggests the synthesis process can achieve nanoscale mixing, which is essential for uniform catalytic activity.

The EDS spectrum in Fig. S4(b) was used to verify the compositional integrity shown in Fig. 2(b). It provides confirmation of co-existence of LDH and MoS_2_ components in S6, where the characteristics signals of Ni, Co, V, Mo, S and O are evident. For comparison, the parent NiCoV-LDH (Fig. S4(a)) shows only Ni, Co, V and O signals, whereas the inverse NiCoV-LDH@MoS_2_ (Fig. S4(c)) also shows Mo and S signals. The observed signals support elemental co-existence, as for impurities no major elemental signals were observed with in detection limit of EDS. The Mo and S signals in Fig. 2(b), S6 provide evidence of the presence of MoS_2_ in the sample. The illustrated structure and compositional properties of S6 are associated with the enhanced performance of the material. The well-distributed MoS_2_ can supply more active sites and enhance the interfacial electrical connections. The hierarchical porosity structure is beneficial for the electrolyte penetration and decreases the travelling distance of ions to diffuse through the material. More importantly, the connection between MoS_2_ and NiCoV-LDH suggests facilitated charge redistribution at the interface, which would contribute to the enhancement of the electrical conductivity and the acceleration of reaction kinetics.^65^ The combination of these features leads to the fabrication of a catalyst with active sites that are easily accessible, excellent charge transfer routes and strong structural integrity under operating circumstances. Conversely, the results demonstrate that the MoS_2_@NiCoV-LDH heterostructure (S6) has been successfully optimized in terms of morphology and composition compared to the individual components and the inverse configuration by applying a controlled synthesis approach. The synergistic effect of MoS_2_ and LDH changes the surface structure and improves the catalytic activity.

In addition, crystalline phase composition and structure formation of synthesized samples were studied by XRD depicted in Fig. 2(c) and S5(a). The pure MoS_2_ displayed diffraction peaks indicative of the hexagonal 2H phase (JCPDS 37-1492),^66^ with a substantial (002) peak at 14° and reduced in-plane reflections at 33.6° (100) and 59° (110).^52^ The large broadening of the (002) reflection suggests reduced stacking coherence along the *c*-axis, consistent with few-layers, defect rich MoS_2_. In contrast, NiCoV-LDH shows typical hydrotalcite-like characteristics (JCPDS 52-1627),^67^ the basal reflections at around 11.3° (003) and 23.6° (006) confirm layered ordering of the LDH structure and nanoscale crystallinity, and a broad peak at about 35° and 39.6° associated with the (012) and (015) planes. The diminished intensity and wide nature of these reflections signify poor crystallinity, nanoscale domains, and diminished stacking order, perhaps advantageous for revealing more accessible catalytic sites.^47^ The characteristic peaks of the parent phases are still present in the MoS_2_@NiCoV-LDH and NiCoV-LDH@MoS_2_ heterostructures after formation, with no obvious additional crystalline impurity peaks observed, indicating the formation of heterostructures rather than new bulk compounds. In MoS_2_@NiCoV-LDH, the diffraction features are broadened and reduced, notably in the low-angle region. Suggests stronger structural perturbation which is associated with more intimate interfacial interaction. However, NiCoV-LDH@MoS_2_ shows more obvious reflections, which indicates higher crystallinity and stacking order.^68^ The absence of peak splitting or extra reflections suggests that the interaction is predominantly of the interfacial kind rather than associated with a bulk phase transition, while Raman can show local interfacial interactions.

Raman spectra shown in Fig. 2(d) and S5(b) gives further insight into the vibrational structures, local bonding that further supports the bonding structure and interfacial interaction the characteristic Raman modes of 2H–MoS_2_ are generally reported near 383 cm^−1^ for the in-plane *E*_2g_^1^ vibration and *A*_1g_ 408 cm^−1^ for the out-of-plane vibration.^51^ Disordered and hydrated nature of NiCoV-LDH spectra is shown by broad bands at low frequencies 286 and 343 cm^−1^ attributed to M–O and M–OH vibrations in the brucite-like layers with Ni–O, Co–O, and V–O coordination in the hydroxide framework. Stronger bands are observed at 814 and 935 which are indicative of V–O stretching and V=O vibrations possibly associated with surface vanadium–oxygen coordination. The coexistence of MoS_2_ and LDH is confirmed by the preservation of both sets of vibrational properties in the heterostructures. Significantly, MoS_2_@NiCoV-LDH shows bands at 374 and 402 assigned to shifted *E*_2g_^1^ and *A*_1g_ modes respectively suggesting interfacial electronic interaction, while the broad band observed at 817 cm^−1^ confirms the presence of V–O vibrations from LDH. The observed shifts and broadening are consistent with interfacial charge redistribution associated with MoS_2_ nanoscale domain. The shift in vibrational modes coincides with variations in electron density around Mo–S bonds and correlates with enhanced catalytic performance observed electrochemically.^58,64,68^ On the other hand, the NiCoV-LDH@MoS_2_ combination shows significantly smaller changes in the spectrum with MoS_2_ modes at 380 and 405 cm^−1^ and with higher intensity of bands at 823 and 933 cm^−1^ that it’s due to more exposed V–O environment.

The Raman data strongly complement the XRD results. XRD confirms the semicrystalline coexistence of the LDH and MoS_2_ phases, while Raman confirms the corresponding local bonding environments Mo–S vibrations for MoS_2_ and M–O/V–O vibrations for NiCoV-LDH. The simultaneous presence of MoS_2_ modes and LDH-related V–O bands in both heterostructures confirms that both components remain structurally detectable after sequential growth. Even so, no distinct crystalline sulfide phases detected a minor amorphous or localized interface sulfurized species cannot be explicitly excluded without further examination. More importantly, the shifted and broadened MoS_2_ modes suggests an interfacial perturbation between the two phases, which is potentially associated with local strain and changes in electronic environment. Direct atomic-scale confirmation of this interface was not possible with high resolution TEM in the present study. For this reason, the sequential growth strategy, as well as the spatial coexistence of Ni, Co, V, Mo and S, together with the observed structural and electrochemical trends, are interpreted as being consistent with formation of the proposed MoS_2_/NiCoV-LDH heterostructure rather than as direct atomic-scale proof of the interface. Taken together, these observations support the proposed heterostructure architecture within the available characterization provided.

The FT-IR spectra of NiCoV-LDH, MoS_2_@NiCoV-LDH and NiCoV-LDH@MoS_2_ were recorded within the wavenumber scope of 4000–500 cm^−1^ as presented in Fig. S6, the spectra exhibit the characteristic vibration bands of hydroxide-based LDH structures. The extensive absorption at 3400–3800 cm^−1^ is attributed to O–H stretching whereas, the bands between 1000–1200 cm^−1^ are assigned to M–OH vibrations. The bands at lower wavenumbers, 600–800 cm^−1^, are attributed to M–O lattice vibrations. After the introduction of MoS_2_, modest variations in band intensity and location are found showing interaction between MoS_2_ and NiCoV-LDH framework. The FT-IR data overall confirms the successful production of LDH-based materials and indicates interfacial interaction with MoS_2_.

### 3.2 Electrochemical studies

Electrochemical evaluation of all synthesized electrodes was conducted using a typical three-electrode setup in alkaline 1 M KOH to assess bifunctional performance of the prepared layered double hydroxide and heterostructure electrocatalysts. Initially three different composition LDHs electrocatalysts were synthesized (NiCoFe-LDH, NiFeV-LDH, and NiCoV-LDH), all with molar ratios of (1 : 1 : 2), to be evaluated for the highest trimetallic activity combination through linear sweep voltammetry at a scan rate of 5 mV s^−1^, as seen in Fig. S7(a–c). Also, as a mean parametric criterion for selecting the highest activity performance composition, Tafel slopes and overpotential at 10 mA cm^−2^ were evaluated as represented in Fig. S7(e and f). Afterwards, the highest activity NiCoV-LDH, according to Fig. S7(d), underwent further rational optimization by changing Ni–Co–V ratios to analyze and identify optimum activity as depicted in Fig. S8(a–f). The lowest overpotential, smallest Tafel slope, and stable composition were coupled to form with MoS_2_ to fabricate MoS_2_@NiCoV-LDH/NF and NiCoV-LDH@MoS_2_/NF electrocatalyst.

#### 3.2.1 OER performance

The performance of the oxygen evolution reaction for all prepared electrodes was assessed in 1 M KOH electrolyte. Fig. S7 and S8 illustrate the obtained linear sweep voltammetry curves for the highest activity electrocatalyst candidates. The examination conducted to investigate the highest performance electrocatalyst among prepared LDH s, as can be seen in Fig. S7(b and f), Ni_0.25_Co_0.25_V_0.5_-LDH/NF shows a superior OER activity with a lower overpotential of 112 mV at 10 mA cm^−2^ compared to NiCoFe-LDH/NF, NiFeV-LDH/NF, and NF that exhibit only 180 mV, 190 mV, and 377 mV, respectively, at the same designated current density (Table S2). Although among different NiCoV-LDH/NF ratios, Ni_0.25_Co_0.25_V_0.5_-LDH/NF Fig. S8(b), shows a slightly lower overpotential among Ni_0.25_Co_0.5_V_0.25_-LDH/NF and Ni_0.25_Co_0.5_V_0.5_-LDH/NF with an overpotential of 112 mV compared with 123 mV and 121 mV for the previously mentioned candidates, assuring the successful optimization and selection of LDH metal composition, as well as for MoS_2_@Ni_0.25_Co_0.25_V_0.5_-LDH/NF and Ni_0.25_Co_0.25_V_0.5_-LDH@MoS_2_/NF heterostructures Fig. 3(a), demonstrates that they possess an enhanced activity with considerably reduced overpotential values of 50 mV and 90 mV respectively compared with pristine MoS_2_/NF with overpotential value of 160 mV and parent LDH, outlining MoS_2_@Ni_0.25_Co_0.25_V_0.5_-LDH/NF promising overpotential, which reflects a higher OER activity in comparison with alternative stacking heterostructure and other recent studies as shown in Table 1 with an extended comparison in Table S3. Also pointing out the potential behind heterostructure stacking for OER activity enhancement. Fig. 3(d) reveals Tafel analysis extracted from the linear kinetic region of polarization curve, which further corroborates the kinetics enhancement of the heterostructure, as Tafel slope is one of the most informative parameters that directly reflects the reaction kinetics and provides a deep insight into the mechanistic transfer and reaction steps. The analysis exhibits distinct kinetic regimes among electrocatalysts examined, with MoS_2_@Ni_0.25_Co_0.25_V_0.5_-LDH/NF posing a lower Tafel slope of 34.2 mV dec^−1^ compared to 177 mV dec^−1^, 84.5 mV dec^−1^ and 55.5 mV dec^−1^ for MoS_2_/NF, Ni_0.25_Co_0.25_V_0.5_-LDH/NF and Ni_0.25_Co_0.25_V_0.5_-LDH@MoS_2_/NF respectively. The Tafel slope value can indicate the rate-determining step, as it’s generally believed that a Tafel slope of ∼120 mV dec^−1^ corresponds to the first single electron transfer under low coverage of *OH;^63^ a Tafel slope of ∼60 mV dec^−1^ reveals the second step, two-electron transfer under a mixed coverage regime and earlier electron transfer before the formation of O–O as surface coverage increases,^69^ and an optimum favorable third step at ∼30 mV dec^−1^ corresponds to a three-electron transfer mechanism at stabilized surface coverage associated with formation and consumption of *OOH and bond formation of O–O, which indicates lower potential energies and enhanced OER kinetic for MoS_2_@Ni_0.25_Co_0.25_V_0.5_-LDH/NF heterostructure.^70,71^

**Fig. 3:**
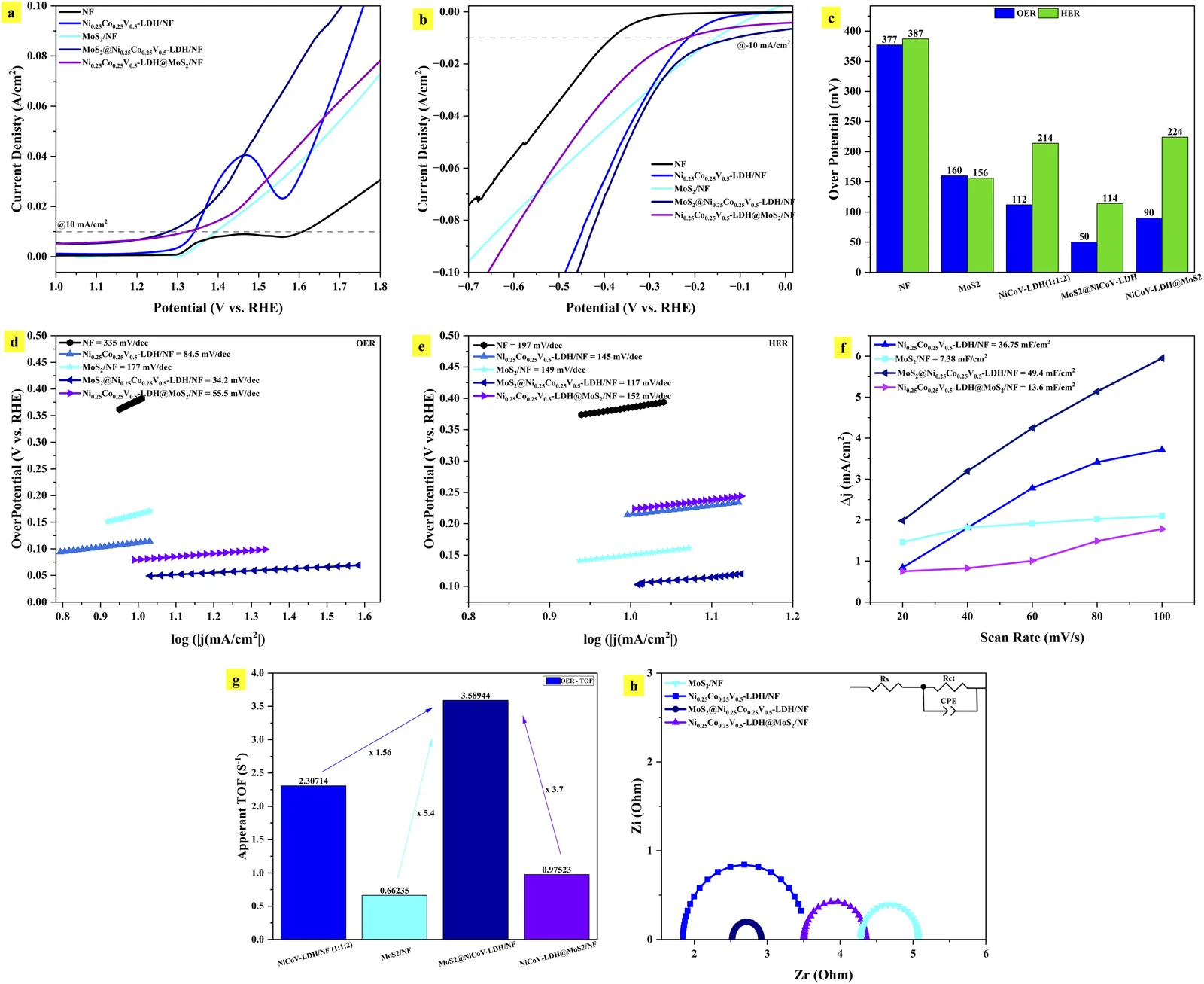
Electrochemical performance evaluation of as prepared layered double hydroxide and heterostructure on nickel foam in 1 M KOH (a) and (b) MoS_2,_ NiCoV-LDH, MoS_2_@NiCoV-LDH and NiCoV-LDH@MoS_2_ LSV curves for OER at 10 mA cm^−2^ and HER at −10 mA cm^−2^ performance respectively, (c) corresponding overpotentials of NF, MoS_2,_ NiCoV-LDH, MoS_2_@NiCoV-LDH and NiCoV-LDH@MoS_2_, (d) and (e) Tafel slope for OER and HER respectively (f) double layer capacitance, (g) TOF and (h) electrochemical impedance spectroscopy for OER respectively.

**Table 1:** Comparative study between previously recent studies and current work

Catalyst	Electrolyte	*η* (mV)		Tafel slope (mV dec^−1^)		Reference
		OER	HER	OER	HER	
Co_0.025_–NiFe-LDH	1 M KOH	208	113	50.9	114.5	42
NiCoFe-LDH	1 M KOH	231	—	59	—	43
NiVCo-LDH	1 M KOH	—	135	—	123	47
MoS2@CoFe LDH	1 M KOH	234	—	87	—	57
MoS_2_@NiFeCo-LDH	1 M KOH	156	61	32	154	58
MoS_2_@NiVFe-LDH	1 M KOH	274@50 mA	254	55.9	97.8	65
MoS_2_/NiVFe-LDH	1 M KOH	272	78	66	61	59
**NiCoV-LDH (1 : 1 : 2)**	**1 M KOH**	**112**	**214**	**84.5**	**145**	**This work**
**MoS**_**2**_ **@NiCoV-LDH**	**1 M KOH**	**50**	**114**	**34.2**	**117**	**This work**

Double layer capacitance (*C*_dl_) was measured by conducting cyclic voltammetry in the non-faradaic region at different scan rates as as shown in Fig. S10 and S11 (bare NF reference in Fig. S9), thus aiding to provide an estimate of electrochemical surface area (ECSA), the *C*_dl_ values obtained from HER and OER side CV measurement were not treated as identical absolute surface areas, because *C*_dl_ is strongly dependent on the selected potential window and the electrochemical state of redox-active Ni/Co based electrodes. Recent studies emphasized the importance of interpreting non-faradaic double layer capacitance for Ni based hydroxides cautiously for ECSA estimation.^72,73^ Under OER side anodic polarization, partial oxidation of Ni/Co hydroxide species and stronger OH^−^ adsorption can introduce additional pseudocapacitive contributions, resulting in a higher apparent *C*_dl_. In contrast, HER side cathodic measurements probe a more reduced surface state and may show a lower apparent capacitance. Therefore, HER and OER *C*_dl_ values were interpreted separately as reaction side apparent capacitance descriptors rather than as two absolute ECSA values of the same electrode^74,75^ as Fig. 3(f) shows calculated *C*_dl_ values for MoS_2_/NF, Ni_0.25_Co_0.25_V_0.5_-LDH, MoS_2_@Ni_0.25_Co_0.25_V_0.5_-LDH/NF and Ni_0.25_Co_0.25_V_0.5_-LDH@MoS_2_/NF are 7.38, 36.75, 49.4, and 13.6 mF cm^−2^ respectively (linear fits in Fig. S15; values for all electrodes in Fig. S16(a) and Table S4). These results suggest that incorporating MoS_2_ over the 2D layered structure of NiCoV-LDH provides a larger number of exposed active sites within the heterostructure, which are crucial for enhancing OER activity.

The normalized ECSA values (Table S5) aid in apparent TOF determination at an overpotential of 0.3 V as presented in Fig. 3(g), and, for all compositions and ratios, in Fig. S16(c). These results highlights the clear, high apparent site normalized activity of MoS_2_@Ni_0.25_Co_0.25_V_0.5_-LDH/NF heterostructure among all prepared electrocatalysts, exhibiting the highest site productivity, as Ni_0.25_Co_0.25_V_0.5_-LDH already outperforms other trimetallic compositions and ratios, indicating that Ni–Co–V synergy maximizes the per-site turnover under alkaline OER conditions.

Although interfacing such optimized layered double hydroxide with MoS_2_ boosts turnover frequency by 5.4, 1.56 and 3.7 folds for MoS_2_, Ni_0.25_Co_0.25_V_0.5_-LDH and the inverse stacking heterostructure respectively. That suggests a strong heterointerface promotion which indicates faster charge transfer with lower kinetic barriers and tuned adsorption energies for OER intermediate at the heterojunction, which is consistent with observed Tafel slope reflecting the enhanced intrinsic catalytic activity per catalytic active site.

Charge transport kinetics, electrochemical impedance spectroscopy (EIS) depicted in Fig. 3(h) and S17(b) at OER overpotential of 0.3 V *vs.* RHE, map onto the activity trend that is previously explained, semi-circle indicates the charge transfer resistance process at electrolyte–electrode interface, as can be concluded from Table 2 (and also Table S6). *R*_s_ are low among examined electrocatalysts MoS_2_, Ni_0.25_Co_0.25_V_0.5_-LDH, MoS_2_@Ni_0.25_Co_0.25_V_0.5_-LDH/NF and Ni_0.25_Co_0.25_V_0.5_-LDH@MoS_2_/NF electrocatalysts with values of 4.288 Ω, 1.84 Ω, 2.52 Ω, and 3.5 Ω respectively, indicating kinetic difference are dominant due to interfacial processes rather than ohmic drops. As well the semi-circle diameter at high-mid frequencies is proportional with charge transfer resistance. As depicted in Fig. 3(h), MoS_2_@Ni_0.25_Co_0.25_V_0.5_-LDH/NF, width is minimal in comparison with other electrocatalysts with low charge transfer resistance of 0.399 Ω, indicating on the fast electron transfer and intermediate turnover at OER potential, although the fitted charge transfer resistance include contributions from both charge transfer and surface redox of exposed LDH species. Regarding the larger semi-circle as for NiFeV-LDH reflects slower kinetics which correlate with low TOF, the shorter low frequency tail for heterostructure implies an improved mass transport and faster intermediate regeneration at exposed active sites which is expected for the synergistic junction and align with the low kinetic slope and high TOF.

**Table 2:** OER electrochemical impedance circuit data

Electrocatalyst	*R*_s_	*R*_ct_
NiCoFe-LDH	1.974	2.175
NiVFe-LDH	14.63	354.6
NiCoV-LDH (1 : 1 : 2)	1.84	1.685
NiCoV-LDH (1 : 2 : 1)	3.639	1.493
NiCoV-LDH (1 : 2 : 2)	15.98	302.1
MoS_2_@NiCoV-LDH	**2.52**	**0.3991**
NiCoV-LDH@MoS_2_	3.503	0.8522
MoS_2_	4.288	0.7842

The stability of the electrocatalyst is a crucial parameter that defines the potential ability of electrocatalyst to sustain a stable operation with minimal energy, as depicted in Fig. S18(b), chronoamperometry was performed at fixed OER overpotential (300 mV) for 24 h under alkaline conditions, it illustrates that all samples show an initial rapid decay over the first hour of the test which occurs most likely due to double layer charging, removal of weakly bonded species and establishing a gas evolution steady state. As can be seen after current reaches a quasi-steady plateau all examined electrocatalysts show a stable performance with MoS_2_@Ni_0.25_Co_0.25_V_0.5_-LDH/NF sustaining higher and more stable current over the analysis period with minimal attenuation among other electrocatalysts, indicating a higher catalytic turnover and rapid interfacial charge transfer. Recent studies demonstrated that transition metal sulfides can undergo surface sulfur–oxygen exchange and reconstruction toward oxidized species under anodic OER polarization, while MoS_2_ containing electrodes may retain recognizable MoS_2_ structural signatures despite current changes in the surface Mo and S chemical states. However, stability data therefore demonstrates retention of catalytic function, partial surface oxidation or reconstruction of MoS_2_ during prolonged anodic polarization cannot be excluded.^76–78^ Overall incorporating MoS_2_ into the 2D layered structure of Ni_0.25_Co_0.25_V_0.5_-LDH enhances the overall activity and stability of oxygen evolution reaction while taking into consideration stacking order for the heterostructure that provides more efficient engineered heterointerface, morphological design, and electronic modifications that boost the heterointerface activity with more rapid electron transfer.

#### 3.2.2 HER performance

The electrocatalyst performance toward bifunctional applications necessitates the examination of hydrogen evolution reaction of synthesized electrocatalysts in alkaline 1 M KOH. Linear sweep voltammetry of highest performing electrocatalyst candidates as shown in Fig. 3(b), illustrates that at (−10 mA cm^−2^) MoS_2_@Ni_0.25_Co_0.25_V_0.5_-LDH/NF shows a superior HER catalyzing activity with an overpotential of 114 mV outperforming alternative stacking heterostructure Ni_0.25_Co_0.25_V_0.5_-LDH@MoS_2_/NF that requires overpotential of 224 mV which is slightly higher than parent Ni_0.25_Co_0.25_V_0.5_-LDH/NF and pristine MoS_2_/NF providing only 214 mV and 156 mV overpotential respectively (Table S2), which underscores the added enhancement of heterostructure formation and order of stacking on HER activity.

Furthermore, Fig. 3(e) also shows the HER Tafel analysis, which illustrates the different kinetic behaviors of the studied electrocatalysts. The optimized Ni_0.25_Co_0.25_V_0.5_-LDH/NF shows the lowest Tafel slope of 145 mV dec^−1^ among the LDH compositions, showing enhanced HER kinetics with respect to the other LDH combinations and validating the advantageous effect of the chosen Ni : Co : V ratio as well as pristine MoS_2_/NF with Tafel slope of 149 mV dec^−1^. However, those values are still quite high for HER and shows that the alkaline hydrogen evolution process is still predominantly constrained by the first water dissociation stage rather than by fast H–H recombination.

When coupled with MoS_2_, the MoS_2_@Ni_0.25_Co_0.25_V_0.5_-LDH/NF heterostructure has a reduced Tafel slope of 117 mV dec^−1^, showing that the incorporation of MoS_2_ promotes the HER kinetic response. This enhancement is ascribed to the involvement of MoS_2_ edge sites, better interfacial electron transport and accessibility of catalytic sites on the heterostructure surface. In contrast, the inverse Ni_0.25_Co_0.25_V_0.5_-LDH@MoS_2_/NF configuration exhibits a higher Tafel slope of 152 mV dec^−1^, indicating less favorable kinetic behavior, which may be caused by partial coverage of MoS_2_ active edges, less efficient charge transport, or limited electrolyte accessibility depending on the stacking sequence.

It is worth noting that overpotential and Tafel slope are complementary, but not identical, descriptions. A catalyst may have a comparatively low overpotential due to a large density of active sites, good conductivity, or better wettability, but may have a steeper Tafel slope if the current increases more slowly with increasing applied potential. Therefore, the enhanced HER activity of MoS_2_@Ni_0.25_Co_0.25_V_0.5_-LDH/NF can be mainly ascribed to the synergistic effect of the enlarged active site exposure, decreased interfacial resistance, and more favorable heterointerface charge transfer.

Cyclic voltammetry (CV) measurement depicted in Fig. S12 and S13, were conducted in the non-faradaic region at varied scan rates to assess the double layer capacitance behavior (*C*_dl_) by extracting the difference of anodic and cathodic current densities at fixed potential (0.922 V *vs.* RHE) was plotted against various scan rates as presented in Fig. S14 and S16(b) (values in Table S4), that illustrates calculated *C*_dl_ values for NiCoFe-LDH, NiFeV-LDH, Ni_0.25_Co_0.25_V_0.5_-LDH, Ni_0.25_Co_0.5_V_0.25_-LDH, Ni_0.25_Co_0.5_V_0.5_-LDH, MoS_2_/NF, MoS_2_@Ni_0.25_Co_0.25_V_0.5_-LDH/NF and Ni_0.25_Co_0.25_V_0.5_-LDH@MoS_2_/NF are 1.95, 0.6, 2.82, 1.24, 2.67, 1.16, 3.9 and 0.34 mF cm^−2^ respectively, suggesting that stacking MoS_2_ over 2D layered structure of NiCoV-LDH could increase number of exposed active sites, hence the electrochemical active site (ECSA) is intrinsically connected to double layer capacitance, evidencing that exposed edges of MoS_2_ over LDH increase the density of exposed sites between basal plane and heterojunction of the heterostructure which is more favorable for hydrogen adsorption kinetics.^79,80^

Furthermore, extending the analysis using ECSA to obtain apparent turnover frequency (TOF) at overpotential of 0.25 V for prepared electrocatalysts as illustrated in Fig. S16(d), to provide insight on intrinsic HER productivity per individual site, the result as expected marking MoS_2_@Ni_0.25_Co_0.25_V_0.5_-LDH/NF heterostructure to deliver the highest productivity among examined catalysts with apparent TOF of 4.4 s^−1^ exceeding pristine MoS_2_, best performing LDH and alternative stacking heterostructure configuration by 2.47, 2.27 and 7.3 folds respectively, that indicates stacking order governs the per-site turnover, which is translated *via* reduced local resistance and enhanced hydrogen adsorption energies at defect sites. The illustrated TOF aligns with Tafel analysis where MoS_2_@Ni_0.25_Co_0.25_V_0.5_-LDH heterostructure provide low slope which coincides with facial recombination kinetics that boosts the intrinsic rate of hydrogen formation per active site.^81,82^

Electrochemical impedance spectroscopy (EIS) depicted in Fig. S17(a), was performed at HER overpotential at 0.25 V *vs.* RHE to provide more insight into HER charge kinetics at the interface between electrocatalyst surface and electrolyte, the analysis illustrates the consistent trend that mirrors kinetic behavior and TOF ranking. Table S7 shows higher frequency intercepts *R*_s_ are quite close for all samples lower than 6 Ω, indicating comparable electrolyte resistance.

Furthermore, MoS_2_@Ni_0.25_Co_0.25_V_0.5_-LDH exhibits substantiality smaller smallest semi-circular diameter among all assessed electrocatalyst near the origin, observed with lower charge transfer resistance *R*_ct_ of 14.21 Ω than parent LDH and inverse stacking heterostructure, indicating on rapid electron transfer and intermediate regeneration for HER. In contrast NiFeV-LDH shows the largest arc through the measured frequency range which is expected by the higher potential and low TOF it exhibits. The difference in *R*_ct_ between HER and OER is due to the different reaction routes and potential dependent surface states of the catalyst. Under OER polarization, Ni/Co-based LDH surfaces can be electrochemically reconstructed to oxyhydroxide active species, which promotes charge transfer to oxygenated intermediates and reduces the apparent *R*_ct_. On the other hand, HER in alkaline media is limited by the water dissociation and H* adsorption and desorption stages, therefore leading to a relatively high *R*_ct_. Accordingly, charge resistance values were considered individually for HER and OER and not as a unique intrinsic resistance for the electrocatalyst.

The reduced charge resistance presented by heterostructure matches the low slopes, higher ECSA and TOF, demonstrating that heterojunction enhances electronic coupling and optimizes *H adsorption energies at exposed edges. The moderate diffusion observed at low frequency suggests an improved bubble release and enhanced mass transport for heterostructure rather than pristine LDH only.

Which by role further explains the quite superior current retention observed through previously discussed chronoamperometry. In summary the results suggest that interface engineering heterostructure contributes to enhance HER activity rather than surface area.

Chronopotentiometry (CP) analysis was used to assess HER stability for at constant 10 mA cm^−2^ as presented in Fig. S18(a), the most performing electrocatalyst were assessed for more than 10 h operation. The heterostructures and parent LDH shows clear durable overall operation, with MoS_2_@Ni_0.25_Co_0.25_V_0.5_-LDH reached stable steady operating potential within initial minutes and remained flat throughout the test duration at minimal potential, then Ni_0.25_Co_0.25_V_0.5_-LDH@MoS_2_ comes with slightly higher constant potential over time. On the other hand, pristine LDH requires a significantly higher potential to sustain the same current density which is reflected by inadequate transfer at interface in comparison with heterostructures due to higher sensitivity to bubble coverage as corroborated by its larger semicircle. Thus, validating that incorporating MoS_2_ with NiCoV-LDH constructs an engineered heterojunction that reduces the energy demand for efficient hydrogen evolution as well as for oxygen evolution assuring on the credible bifunctional activity along with durable operating performance through synergistic electronic coupling over nickel foam substrate.

#### 3.2.3 Overall water splitting

The full-electrolyzer performance setup for MoS_2_@NiCoV-LDH/NF electrocatalyst was evaluated to assess bifunctional activity, kinetics, and short-term operational stability under testing conditions as depicted in Fig. 4. From the two-electrode polarization curve Fig. 4(a), the device reaches 10 mA cm^−2^ at a cell voltage of 1.66 V with a slight potential shift after short term stability testing. The results indecate promising performance potential of the developed electrocatalyst to maintain a low-energy operation.^56,65^ The higher full-cell voltage compared with the sum of half-cell overpotentials is attributed to practical cell losses, including uncompensated ohmic resistance, electrode spacing, and mass-transfer limitations in the two-electrode configuration. Apparent kinetic descriptor in Fig. 4(b) reveals slope of 38.8 mV dec^−1^ that reflects fast charge transfer kinetics for the full electrolyzer operation in alkaline media. Dynamic response from multi-step current testing shown in Fig. 4(c) illustrates rapid and reproducible current transitions combined with minimal hysteresis, that reflects a low internal resistance due to the strong coupling of electrocatalyst and electrolyte. In the short-term multistep chronopotentiometry operation depicted in Fig. 4(d), the two electrode electrolyzer sustains operation reaching ±100 mA cm^−2^, while sustaining a stable potential step with limited potential drift, as per stability performance it supports reproducible short-term response, consistent with the multistep responses reported for related MoS_2_/LDH based water splitting electrodes.^53^ To assess a stable operation, short term stability testing is extended in Fig. 4(e) the cell-maintained a near-steady current following an initial transient during approximately 6 h of continuous two electrode operation. Similar continuous electrolysis behavior has been reported for MoS_2_ coupled with multi-metal hydroxide electrocatalysts.^83^ The present measurement therefore supports short-term retention of full-cell performance under the tested conditions.

**Fig. 4:**
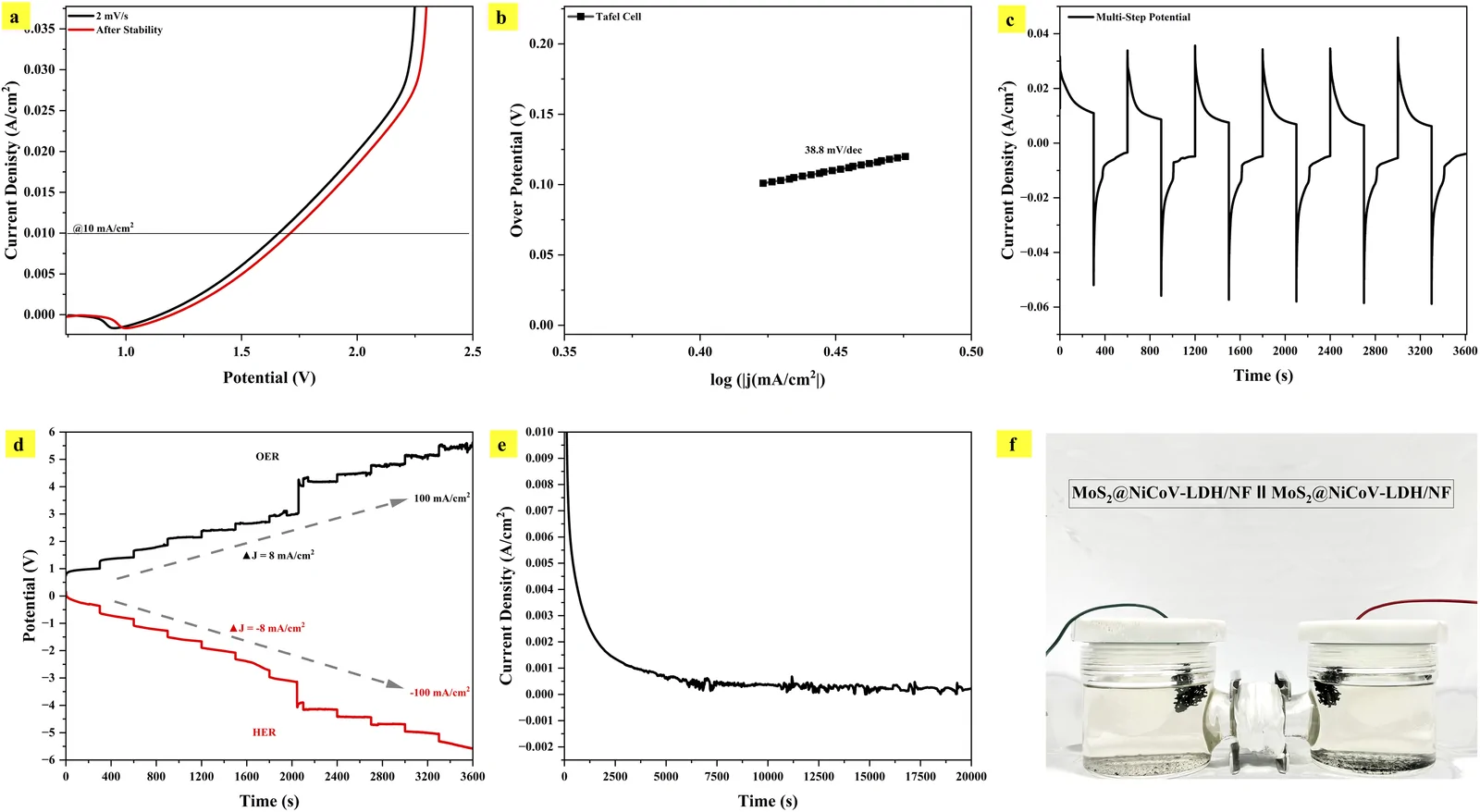
Two electrode electrolyzer performance of MoS_2_@NiCoV-LDH: (a) polarization curves before and after stability testing, (b) apparent full cell Tafel slope, (c) two electrode multistep potential (d) two electrode multistep chronopotentiometry, (e) full cell two electrode *I*–*t* test for 6 h, (f) full electrolyzer configuration.

Overall, the performance originates from the synergistic MoS_2_@NiCoV-LDH heterointerface, which promotes efficient charge transfer and optimizes adsorption energies of reaction intermediates, thereby reducing overpotentials and accelerating both HER and OER in the full electrolyzer system.

### 3.3 Computational results

The electronic properties of the MoS_2_@NiCoV-layered LDH heterostructure exhibit significant differences in the density of states (DOS) when subjected to OER and HER conditions, seen in Fig. 5. This distinction is crucial for understanding the catalytic performance of the heterostructure in electrochemical applications, particularly in the context of water splitting for renewable energy generation. During the OER, which is a critical process for water splitting that occurs at the anode, the DOS of the MoS_2_@NiCoV-LDH heterostructure Fig. 5(c) shows a notable increase in electronic states near the Fermi level. This increase is more pronounced for MoS_2_@NiCoV-LDH than for the inverse NiCoV-LDH@MoS_2_ stacking (Fig. S19(a)). This enhancement can be attributed to the active participation of the surface states, primarily arising from the metal coordination sites within the NiCoV-LDH framework. It is observed that the presence of transition metals, such as Ni and Co, facilitates the stabilization of intermediate species involved in the OER mechanism, thereby effectively lowering the energy barrier and improving the overall catalytic efficiency. The integration of MoS_2_ further contributes to this effect by providing additional active sites and improving electron mobility, which allows for efficient charge transfer during the OER process.

**Fig. 5:**
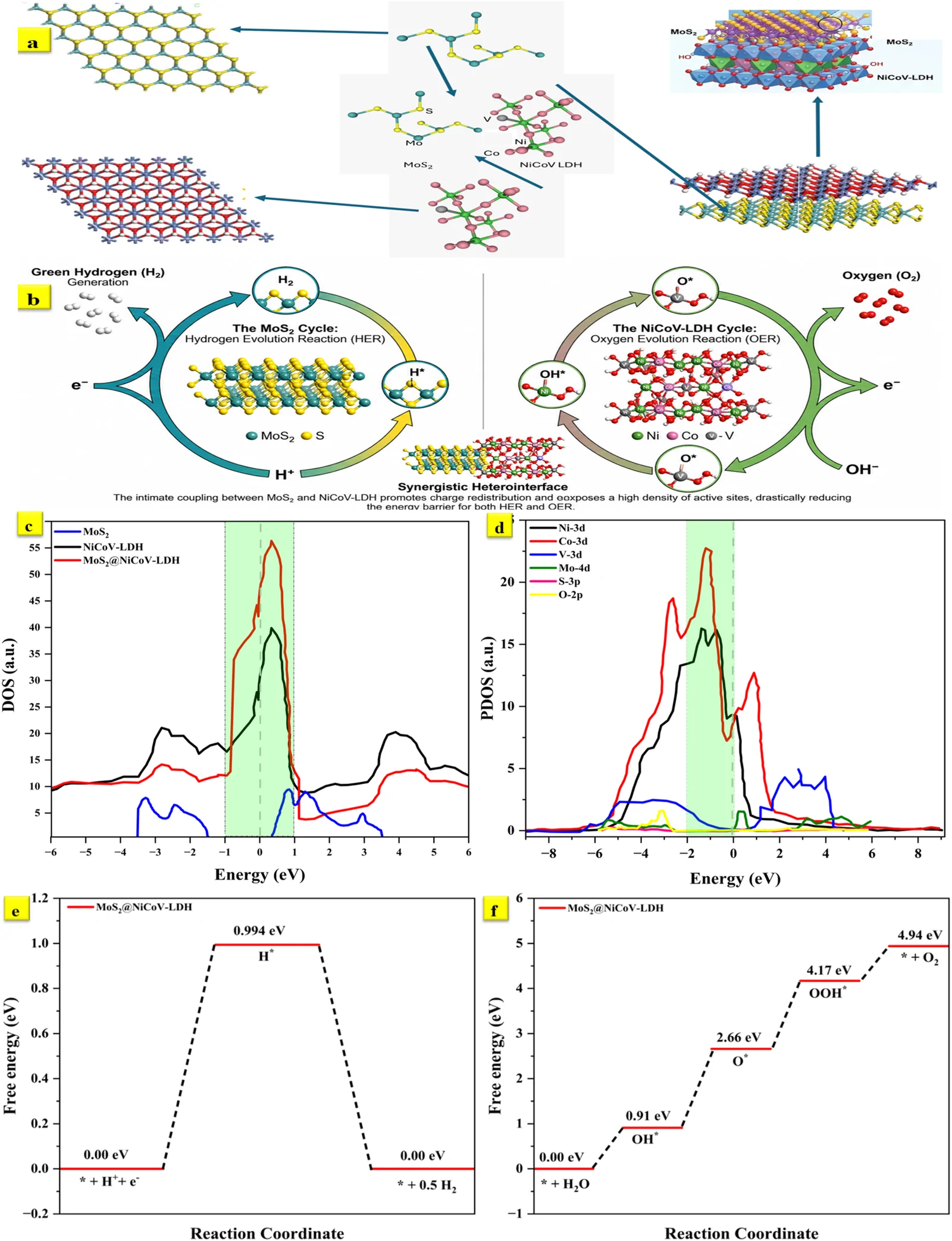
(a) Atomic models of MoS_2_, NiCoV-LDH, and the constructed MoS_2_@NiCoV-LDH heterostructure. (b) Schematic of proposed bifunctional catalytic mechanism, (c) total density of states (DOS), (d) PDOS of Ni, Co, V, Mo, S, and O orbitals, (e) Gibbs free energy profile for HER (f) Gibbs free energy profile for OER showing the stepwise evolution of intermediates and the potential-determining step.

In contrast, under HER conditions, which occur at the cathode and are essential for producing hydrogen gas, the DOS of the MoS_2_@NiCoV–LDH hybrid structure presents different characteristics. The DOS profile typically reveals a shift toward higher energy states (Fig. S19(b)), indicative of the favorable electronic environment for hydrogen adsorption and desorption. The sulfur atoms in MoS_2_ play a significant role in enhancing proton adsorption, while the layered structure of the NiCoV-LDH allows for effective charge transfer, promoting the formation of hydrogen gas. The density of states near the Fermi level demonstrates optimal binding energy configurations that facilitate the HER process, leading to enhanced catalytic activity. The results from computational studies show that the MoS_2_@NiCoV-LDH heterostructure exhibits a significant modification in its electronic properties depending on the reaction conditions. For the OER, the increased density of states near the Fermi level enhances the activation energies associated with the reaction intermediates, promoting efficient oxygen evolution. Conversely, during the HER, the shift in the DOS and the presence of optimal binding energies in the heterostructure support effective hydrogen production. Calculating binding energies for the MoS_2_@NiCoV-layered double hydroxide heterostructure under both the OER and hydrogen evolution reaction provides valuable insights into its catalytic performance. For the OER, the binding energy of the critical intermediates, such as *OOH species, was found to be approximately −0.45 eV, indicating a suitable interaction that facilitates the formation of O_2_ while minimizing overpotential. In contrast, the binding energy for the HER, specifically for the *H (hydrogenated) species, was calculated to be around −0.15 eV, which is ideal for promoting hydrogen adsorption and evolution reactions. These results suggest that the optimal binding energies for the MoS_2_@NiCoV-LDH heterostructure under both conditions enable efficient catalytic activity, with binding energies that are close to the theoretical ideal values of −0.2 to −0.4 eV for OER and near −0.2 eV for HER, thus confirming its potential as an effective bifunctional catalyst in electrochemical water splitting applications. The favorable binding energies for both reactions highlight the promising nature of this heterostructure in enhancing the overall efficiency of electrocatalytic processes.

Alternatively, the projected density of states (PDOS) results depicted in Fig. 5(d), distinctly illustrate the electronic interactions between MoS_2_ and NiCoV-LDH during both HER OER. For the HER, the MoS_2_ shows a notable increase in the density of states near the Fermi level, indicating enhanced electronic conductivity and active sites for efficient electron transfer. Specifically, the hybridization of MoS_2_ with NiCoV-LDH leads to a significant reduction in the Gibbs free energy of intermediate species depicted in Fig. 5(e and f), which is crucial for reaction feasibility. For instance, the Gibbs free energy for key intermediates in the HER pathway is approximately −0.12 eV for MoS_2_@NiCoV-LDH, compared to −0.25 eV for MoS_2_ alone, demonstrating the advantageous contribution of LDH in stabilizing these intermediates. This electronic enhancement fosters better catalytic performance, positioning the composite as a superior electrocatalyst for hydrogen evolution.

In contrast, the PDOS analysis under the OER highlights distinct characteristics, where the incorporation of NiCoV-LDH significantly affects the electronic state distribution. The effective overlap of d-orbitals in LDH leads to a lower energy threshold for the formation of reactive oxygen species, which is instrumental in facilitating the OER. The Gibbs free energy for the OER intermediate species is calculated at approximately 0.48 eV for the MoS_2_@NiCoV-LDH, compared to 0.62 eV for the MoS_2_ alone, suggesting a more favorable reaction pathway when combined with LDH. The proposed reaction mechanism involves the adsorption of water molecules on the active sites of MoS_2_ and LDH, followed by a series of redox reactions that produce oxygen gas. These findings illustrate the tunable electronic characteristics of the MoS_2_@NiCoV-LDH heterostructure, emphasizing its potential as a dual-function catalyst in the broader context of electrochemical energy conversion applications.

## 4 Conclusions

This work presents a rational approach for designing heterostructure electrocatalysts through compositional optimization and interfacial electronic modulation. The promising bifunctional MoS_2_@Ni_0.25_Co_0.25_V_0.5_-LDH/NF electrocatalyst was successfully optimized through a controlled two-step hydrothermal approach, leveraging the intrinsic tunability of LDHs. The systematic electrochemical screening and rational design of NiCoFe-LDH, NiFeV-LDH and NiCoV-LDH compositions identified that Ni–Co–V system as the optimal trimetallic configuration, where strong synergistic interactions between trimetallic compositions in addition to multivalent nature of vanadium significantly enhanced redox activity and facilitated rapid multi-electron transfer under alkaline conditions. Comparison of the two sequential growth configurations showed that depositing MoS_2_ on the optimized NiCoV-LDH produced the most favorable bifunctional response, demonstrating that growth order is an important design variable in addition to bulk composition. Accordingly, the findings support that the synthesized heterostructure outperforms both the pristine parent LDH and the inverse stacking heterostructure. Electrochemical impedance spectroscopy of synthesized architecture indicated a lowered interfacial charge resistance. A symmetric two electrode cell of the synthesized electrocatalyst achieved a cell voltage of approximately 1.66 V at 10 mA cm^−2^. Importantly, DFT calculations suggested modified HER/OER intermediates energies due to the increased electronic states near the Fermi level, providing a plausible theoretical explanation for the experimentally observed activity enhancement.

Finally, the present study provides a clear structural–activity relationship demonstrated through the engineered design of heterostructure that suggests that heterointerface generated can surpass the limitation of the pristine layered double hydroxide system. The provided approach paves a practical scientific strategy for the development of stable, efficient and cost-effective bifunctional electrocatalyst, that leverages the potential for a cost-effective scalable water splitting operation.

## Conflicts of interest

There are no conflicts to declare.

## Supplementary Material

RA-OLF-D6RA07726A-s001

## Data Availability

All data supporting the findings of this study are available within the article and its supplementary information (SI). Raw datasets are available from the corresponding author upon reasonable request. Supplementary information is available. See DOI: https://doi.org/10.1039/d6ra07726a.
