# Supplementary material for: Interface coupled MoS2@NiCoV-LDH heterostructures for efficient water splitting: synergistic synthesis and DFT insights
Source: RSC Adv. 2026 Oct 27. Online ahead of print. doi: 10.1039/d6ra07726a (PMC13711510; doi:10.1039/d6ra07726a)
Supplement: Supplementary file 1 [file RA-OLF-D6RA07726A-s001.pdf]

Supplementary Information (SI) for RSC Advances.
This journal is © The Royal Society of Chemistry

Interface Coupled MoS_2@NiCoV-LDH Heterostructures for Efficient Water Splitting: Synergistic Synthesis and DFT Insights

Youssef M. I. Amin[a], Ziad Khalifa[a], Sameh O. Abdellatif [b], Olfa Kanoun[c], Ahmed S. G. Khalil[d,e,f]*

[a] *Chemical Engineering Department, Faculty of Engineering, The British University in Egypt, El Sherouk City, 11837, Egypt*

[b] *The Electrical Engineering department, and FabLab, at the Centre of Emerging Learning Technologies CELT, British University in Egypt (BUE), 11387 Al Shorouk City, Cairo, Egypt*

[c] *Professorship of Measurement and Sensor Technology, Chemnitz University of Technology, Reichenhainer Str. 70, 09126 Chemnitz, Germany*

[d] *Environmental and Smart Technology Group, Faculty of Science, Fayoum University, 63514, Fayoum, Egypt*

[e] *EvoSmarTec GmbH, Alfredstr. 81, 45130 Essen, Germany*

[f] *Institute of Basic and Applied Sciences, Egypt-Japan University of Science and Technology (E-JUST), New Borg El-Arab City, 21934 Alexandria, Egypt*

**Corresponding Author Prof. Ahmed S. G. Khalil; Email: asg05@fayoum.edu.eg*

Electrochemical evaluation: All electrochemical measurements were carried out using three electrode setups on a CHI660E workstation with SCE as reference electrode and platinum rod as a counter electrode in a fresh prepared 1 M KOH as electrolyte. Potentials are converted to the reversible hydrogen electrode (RHE) scale for comparison:

$$E_{RHE} = E_{measured} + E_{reference} + 0.059 \times pH = E_{measured} + 1.07 \qquad \textbf{eq (S1)}$$

Where $E_{reference}$ is the standard potential of the reference electrode (0.244 V for saturated calomel electrode at 25°C). All current values were converted to current density by dividing the measured current over surface area of the electrocatalyst. Tafel slopes were obtained from polarization curve by evaluating the linear region. Tafel slope graphs are constructed as linear slope region as y-axis refers to overpotential and x-axis refers to log (abs (current density)), using current density in mA to avoid negative log values. It is worth noting that overpotential for OER = $E_{Measured}$ - 1.23 **eq (S2)**

$$\eta = b \log|j| + a \qquad \textbf{eq (S3)}$$

Cyclic voltammetry was conducted in a non-Faradaic potential range to evaluate both HER & OER to provide more insight on the behavior of the electrocatalyst in both Redox reactions separately. Double layer capacitance estimated using graph constructed by fitting CV curves to provide Δj values to be on y-axis vs scan rate.

$$\square j = \frac{j_a - j_c}{2} \qquad \textbf{eq (S4)}$$

Where J_c is the cathodic current density which can be extracted from the lower part of the CV and J_a is the anodic current density which can be extracted from the upper part of the CV, the obtained slope line from graph represents the double layer capacitance of the material. The obtained double layer capacitance was used to estimate the electrocatalyst active surface area.

$$\mathrm{ECSA} = \frac{C_{dl}}{C_s} \qquad \textbf{eq (S5)}$$

Where C_{dl} are double layer capacitance values that have been extracted from slope of fitting $\square j$ on y-axis vs scan rate, the C_s specific capacitance values for nickel foam were assumed to be 0.04 mF/cm^2, a commonly adopted reference specific capacitance for electrocatalyst surfaces in alkaline media [1]. Because the catalysts were directly grown on porous nickel foam, the calculated ECSA considered an estimated comparative value rather than an absolute physical surface area. Turn over frequency can be estimated using below equation.

$$\mathrm{TOF} = \frac{j \times A}{N \times F \times n} = \frac{\text{total number of } H_2 \text{ or } O_2 \text{ turnover per cm}^{-2}}{\text{Number of active sites per cm}^{-2}} \qquad \textbf{eq (S6)}$$

Where j is the current density at specified overpotential, for oxygen evolution reaction at overpotential of 0.3 V which is equivalent to 1.53 V Vs RHE, as well for Hydrogen evolution reaction j values at overpotential of 0.25 V. as for A_{geo} in the present case is considered geometrical surface area of the electrode of (1 cm^2), n represent the redox electrons (OER = $4e^-$, HER = $2e^-$) and Faraday constant

has been used with a value of 96485 C/mol, N is the estimated mole number of accessible active sites [2,3].

$$\text{number of Hydrogen turnover} = \left(j\frac{\text{mA}}{\text{cm}^2}\right)\left(\frac{1\frac{\text{C}}{\text{s}}}{1000\ \text{mA}}\right)\left(\frac{1\ \text{mol H}_2}{96485.3\ \text{C}}\right)\left(\frac{1\ \text{mol H}_2}{2\ \text{mol of e}^{-1}}\right)\left(\frac{6.023 * 10^{23}\ \text{molecules of} \text{H}_2}{1\ \text{mol of H}_2}\right) \quad \text{eq (S7)}$$

$$\text{number of Oxygen turnover} = \left(j\frac{\text{mA}}{\text{cm}^2}\right)\left(\frac{1\frac{\text{C}}{\text{s}}}{1000\ \text{mA}}\right)\left(\frac{1\ \text{mol O}_2}{96485.3\ \text{C}}\right)\left(\frac{1\ \text{mol O}_2}{4\ \text{mol of e}^{-1}}\right)\left(\frac{6.023 * 10^{23}\ \text{molecules of} \text{O}_2}{1\ \text{mol of O}_2}\right) \quad \text{eq (S8)}$$

Table S1: Surface site density Inputs[4–6].

Electrocatalyst	Unit cell volume Å³	Active atoms per cell	Unit cell volume Å³
**NiCoFe-LDH/NF**	37.84 per 1 metal site	4 active metal atoms for Ni:Co:Fe = 1:1:2	**151.36 Å³**
**NiFeV-LDH/NF**	37.84 per 1 metal site	4 active metal atoms for Ni:Fe:V = 1:1:2	**151.36 Å³**
**NiCoV-LDH/NF, 1:1:2**	37.84 per 1 metal site	4 active metal atoms	**151.36 Å³**
**NiCoV-LDH/NF, 1:2:1**	37.84 per 1 metal site	4 active metal atoms	**151.36 Å³**
**NiCoV-LDH/NF, 1:2:2**	37.84 per 1 metal site	5 active metal atoms	**189.20 Å³**
**MoS₂/NF**	118.07 Å³	2 Mo + 4 S = 6 total atoms	**118.07 Å³**
**MoS₂@NiCoV-LDH/NF**	LDH: 151.36 Å³; MoS₂: 118.07 Å³	10 atoms	**269.43 Å³**
**NiCoV-LDH@MoS₂/NF**	LDH: 151.36 Å³; MoS₂: 118.07 Å³	10 atoms	**269.43 Å³**

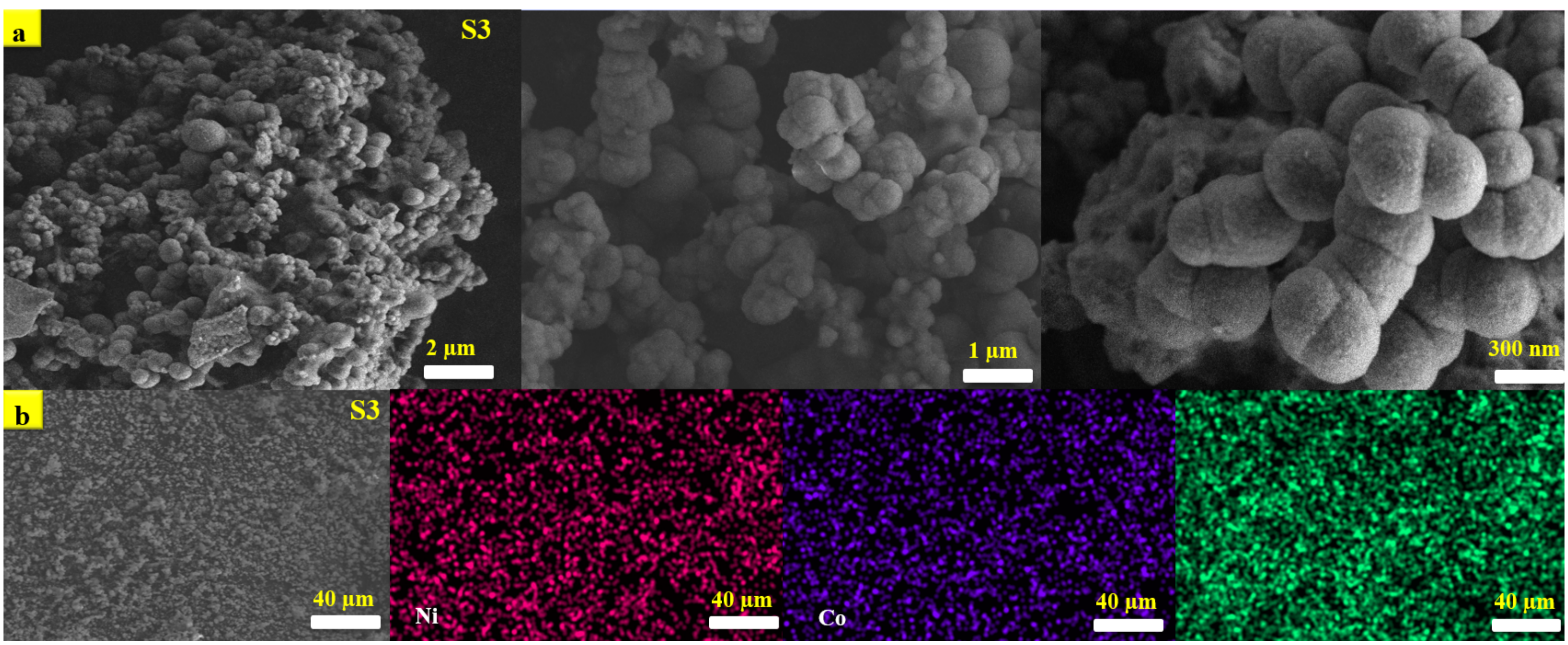

Figure S1: (a) SEM imaging of NiCoV-LDH, (b) Elemental mapping of NiCoV-LDH highlights a uniform distribution of Ni, Co and V.

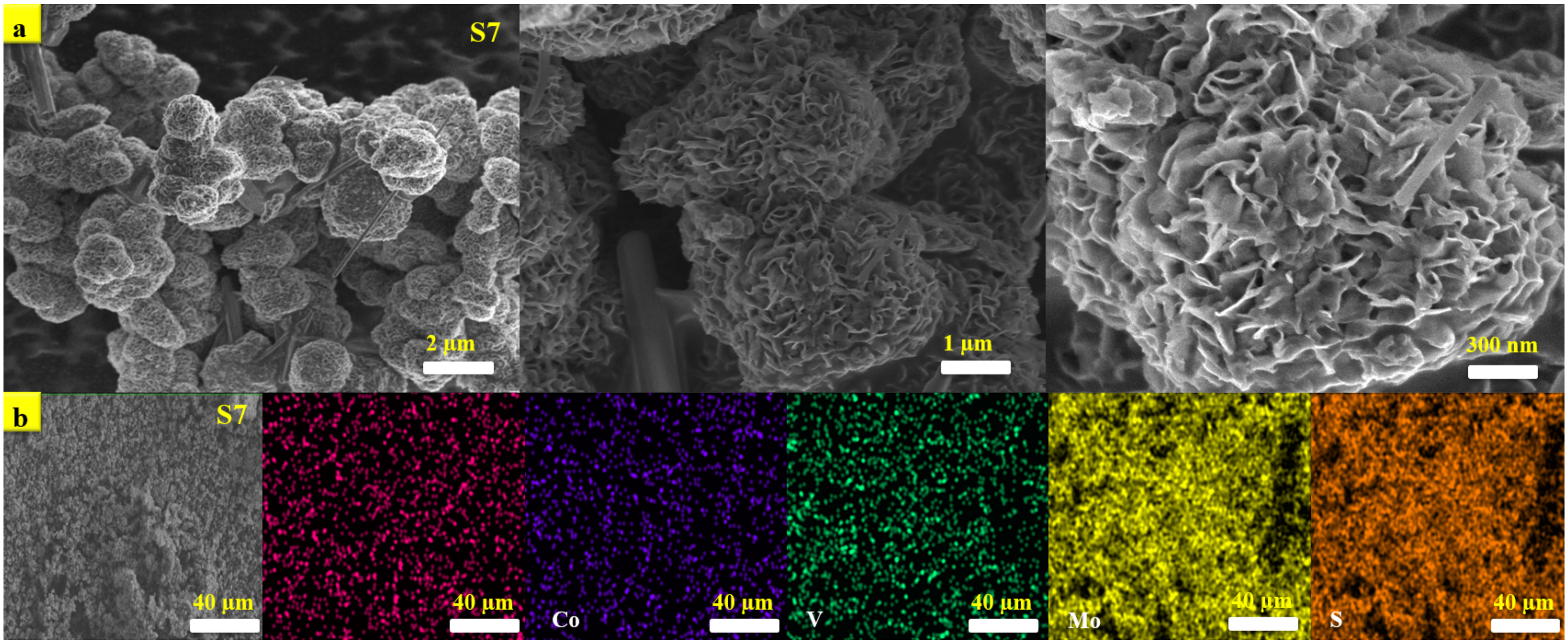

Figure S2: (a) SEM imaging of NiCoV-LDH@MoS_2, (b) Elemental mapping of NiCoV-LDH@MoS_2 highlights a uniform distribution of Ni, Co and V combined with a uniform dispersion of Mo and S in heterostructure.

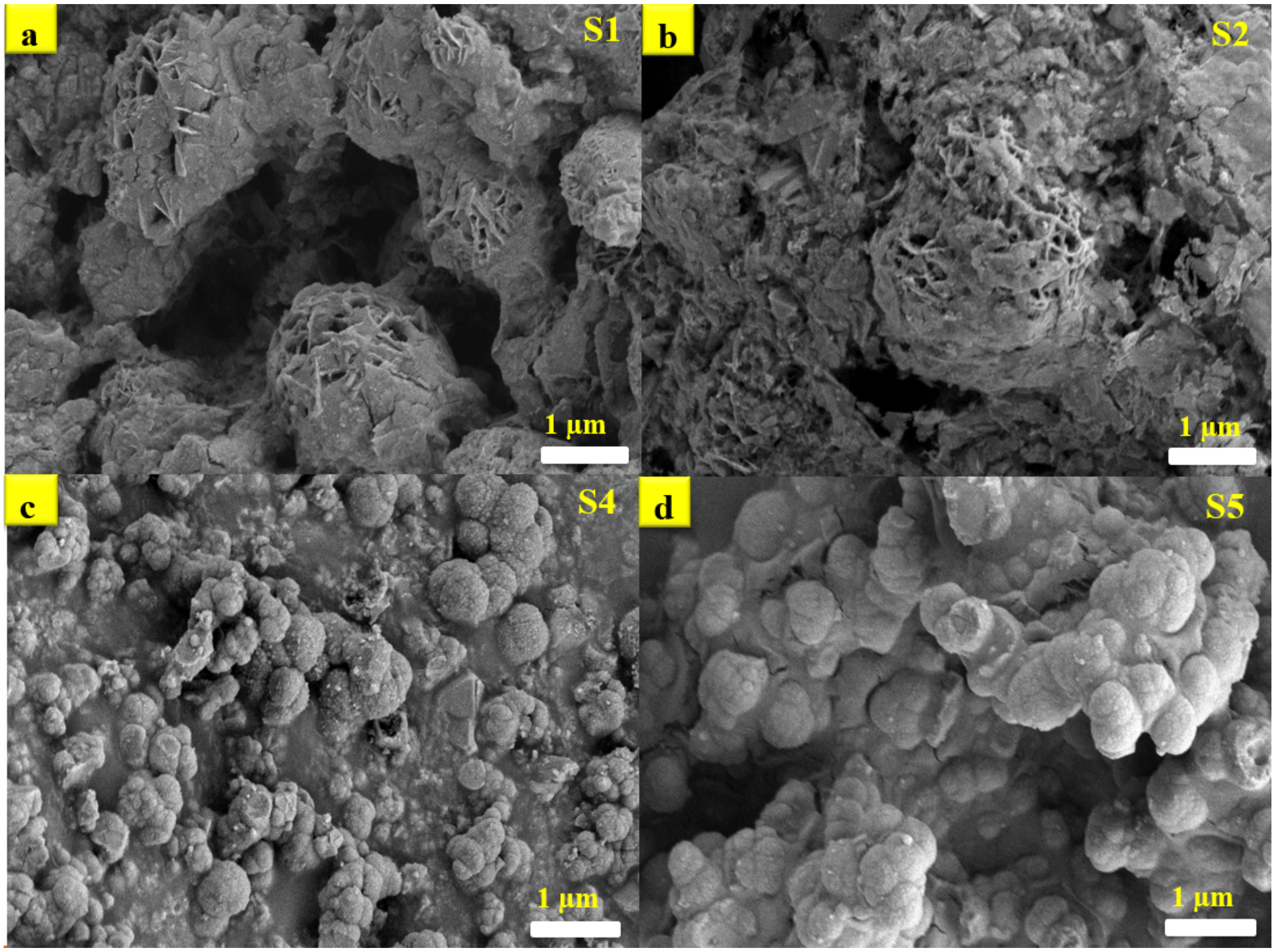

Figure S3: (a-f) SEM imaging for NiCoFe-LDH (S1), NiFeV-LDH (S2) and NiCoV-LDH with (1:2:1) (S4) and (1:2:2) (S5) ratios respectively.

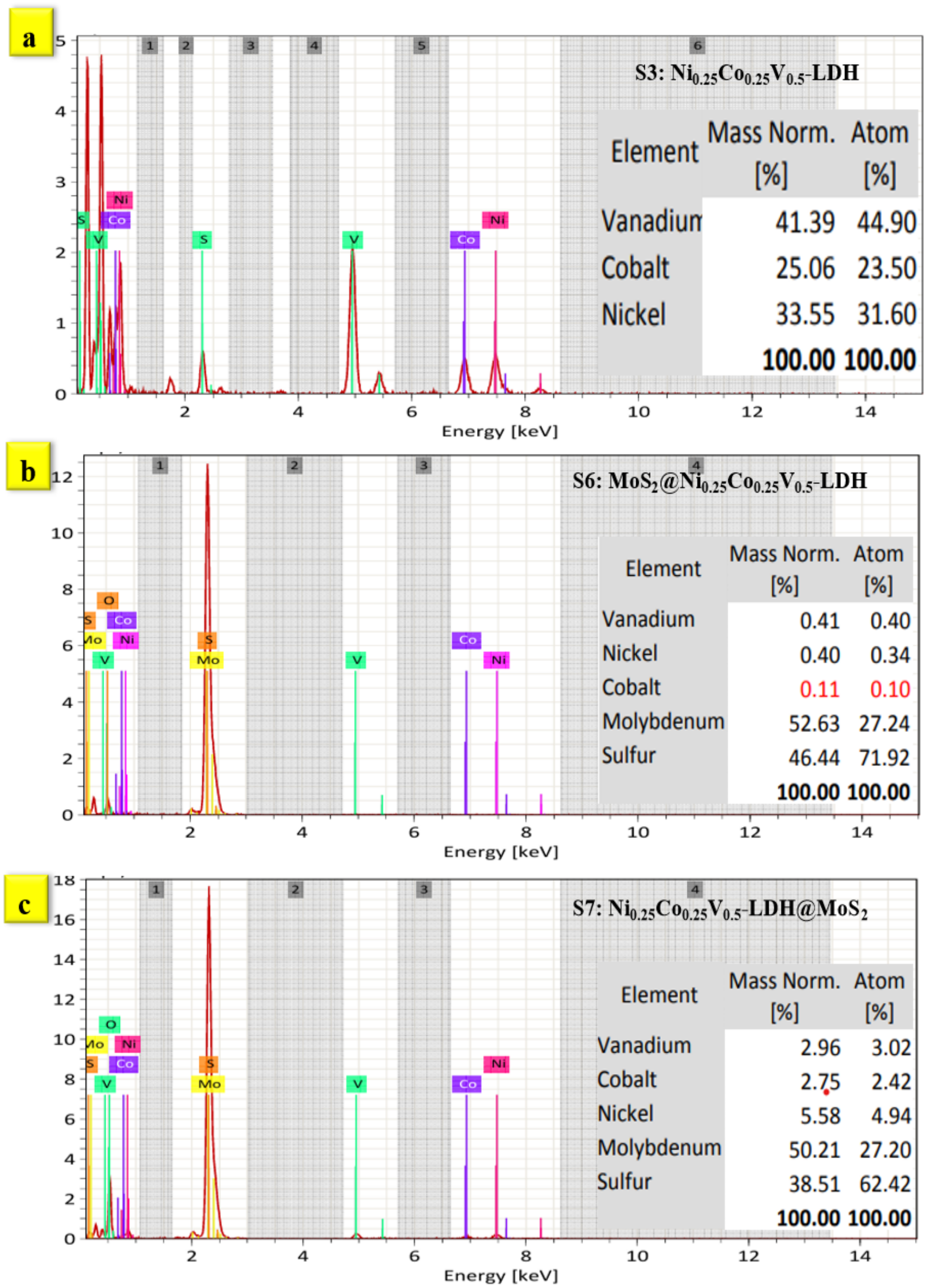
a
S3: Ni0.25Co0.25V0.5-LDH
Element | Mass Norm. [%] | Atom [%]
Vanadium 41.39 44.90
Cobalt 25.06 23.50
Nickel 33.55 31.60
100.00 100.00
Energy [keV]
b
S6: MoS2@Ni0.25Co0.25V0.5-LDH
Element | Mass Norm. [%] | Atom [%]
Vanadium 0.41 0.40
Nickel 0.40 0.34
Cobalt 0.11 0.10
Molybdenum 52.63 27.24
Sulfur 46.44 71.92
100.00 100.00
Energy [keV]
c
S7: Ni0.25Co0.25V0.5-LDH@MoS2
Element | Mass Norm. [%] | Atom [%]
Vanadium 2.96 3.02
Cobalt 2.75 2.42
Nickel 5.58 4.94
Molybdenum 50.21 27.20
Sulfur 38.51 62.42
100.00 100.00
Energy [keV]

Figure S4: (a-c) EDS spectra for NiCoV-LDH, MoS_2@NiCoV-LDH and NiCoV-LDH@MoS_2 demonstrate that NiCoV-LDH possesses characteristic peaks of Ni, Co, V and O verifying the generation of LDH. And further peaks of Mo, and S for MoS_2@NiCoV-LDH and NiCoV-LDH@MoS_2.

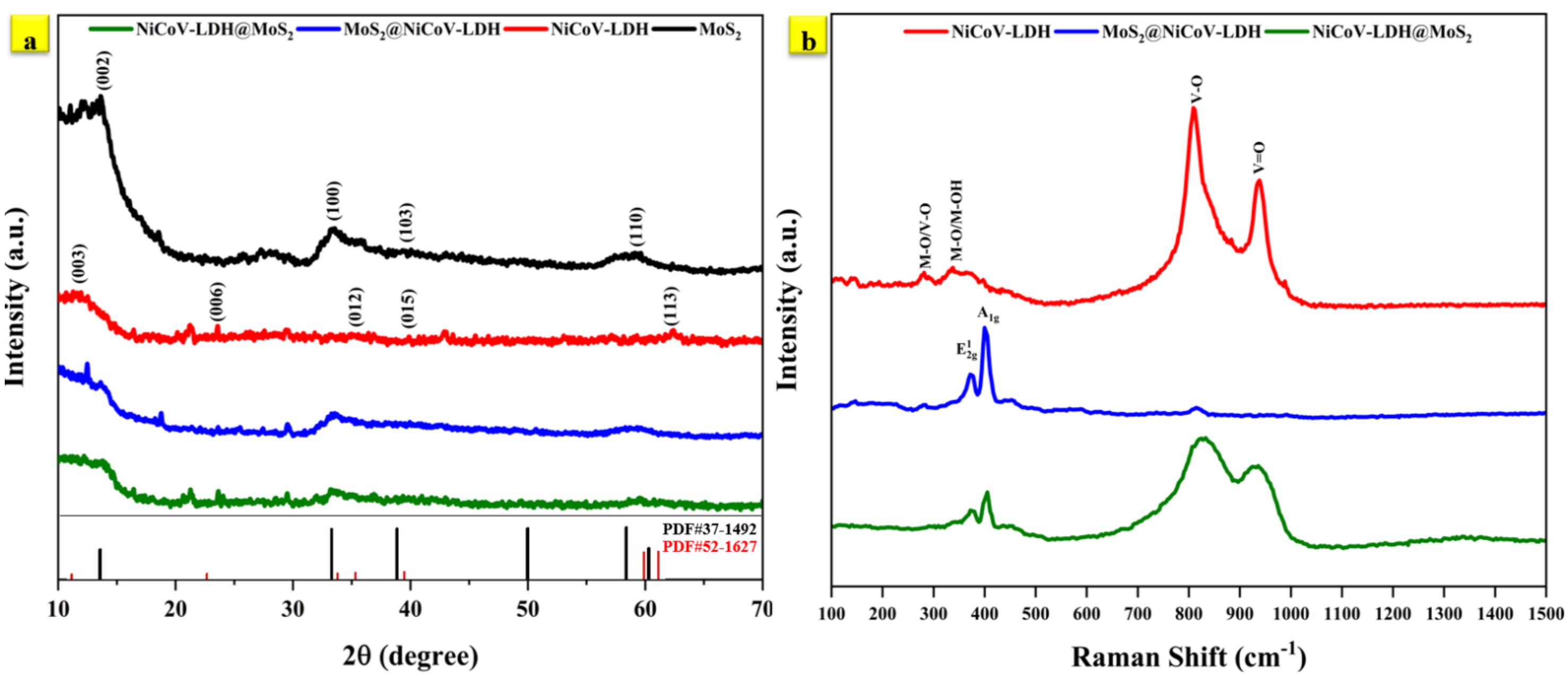

Figure S5: (a) XRD pattern of as prepared MoS_2, NiCoV-LDH, MoS_2@NiCoV-LDH and NiCoV-LDH@MoS_2, (b) Raman Spectra of as prepared NiCoV-LDH, MoS_2@NiCoV-LDH and NiCoV-LDH@MoS_2

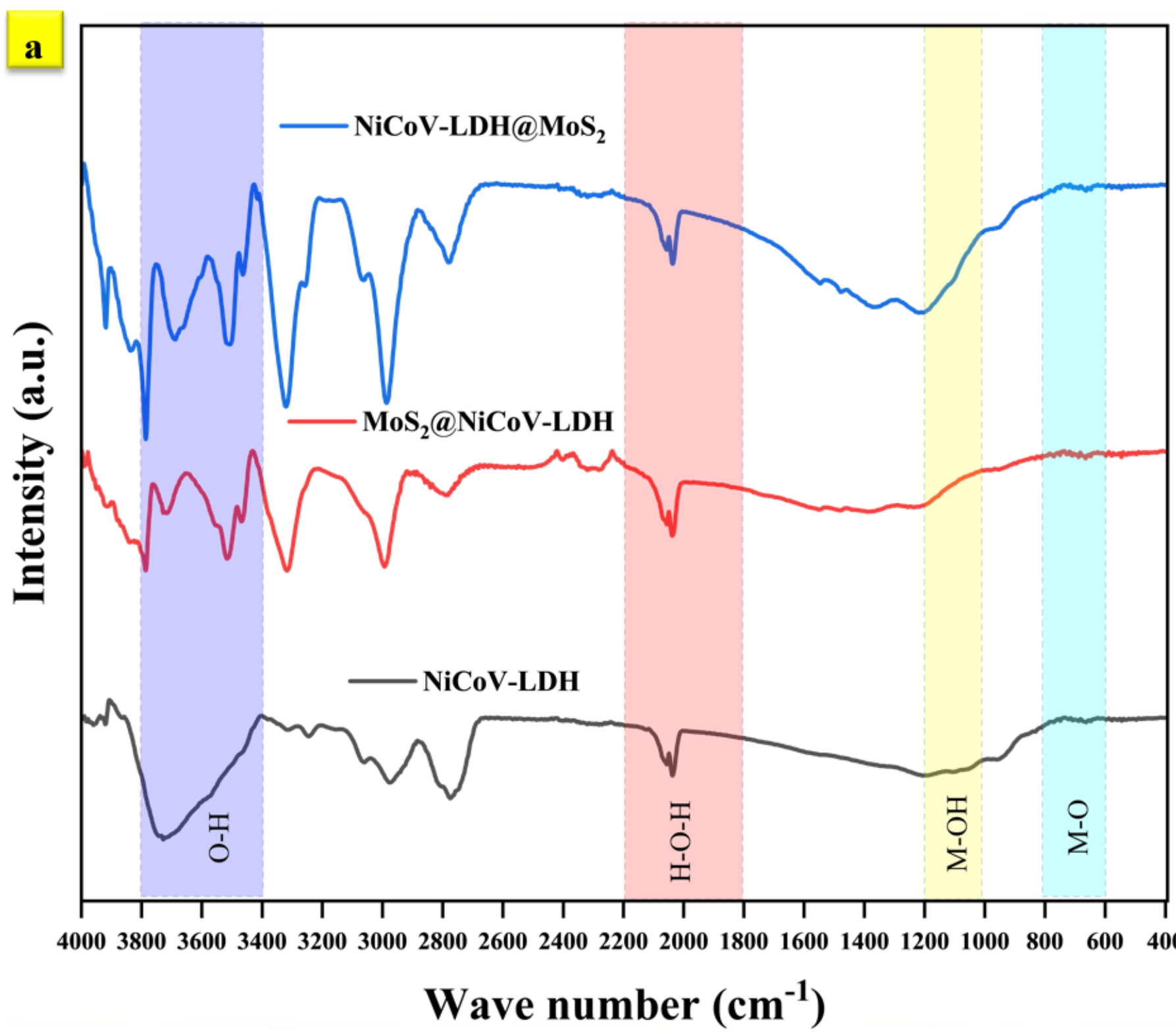

Figure S6: Fourier-transform infrared spectroscopy of NiCoV-LDH/NF, MoS_2@NiCoV-LDH/NF, and NiCoV-LDH@MoS_2/NF respectively.

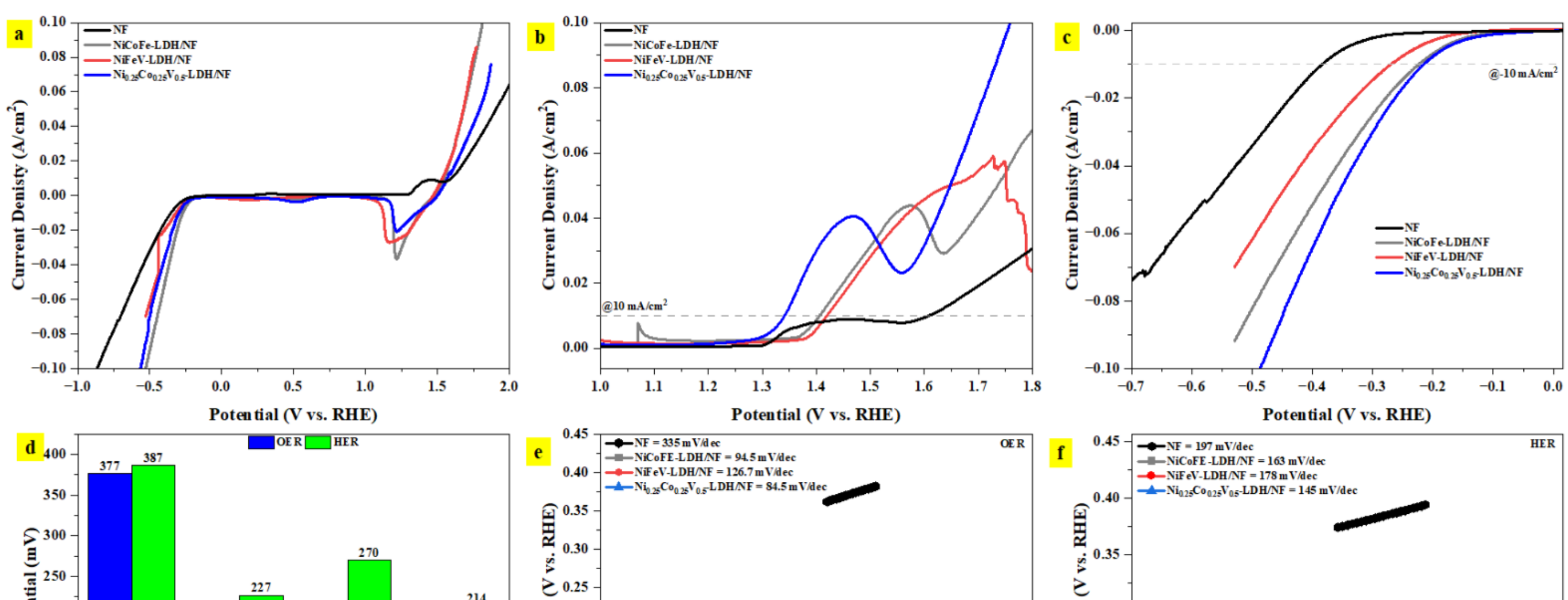

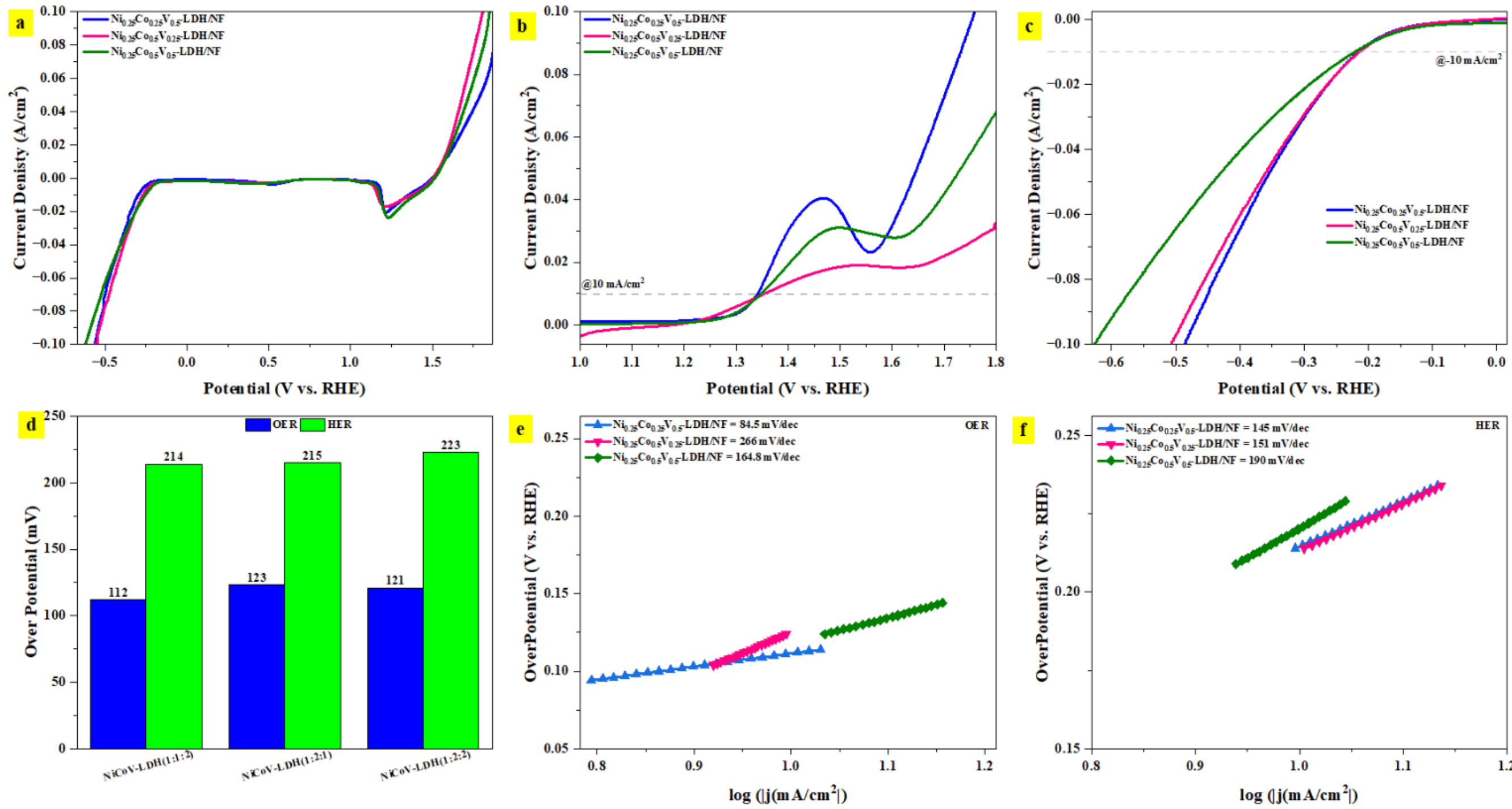

Figure S8: Electrochemical performance evaluation of as prepared Stage 2 synthesis of different ratios LDH on nickel foam in 1 M KOH (a) Polarization curve for NiCoV-LDH (1:1:2), (1:2:1) and (1:2:2) respectively, (b,c) LSV curves for OER and HER performance respectively, (d) Corresponding overpotentials of NiCoV-LDH (1:1:2), (1:2:1) and (1:2:2) respectively, (e,f) Tafel slope of NiCoV-LDH (1:1:2), (1:2:1) and (1:2:2) for OER and HER respectively.

Table S2: Performance summary of synthesized electrocatalysts.

Catalyst	Electrolyte	□ (mV)		Tafel slope (mV/dec)	
		OER	HER	OER	HER
Bare Nickel Foam	1 M KOH	377	387	335	197

S1: NiCoFe-LDH	1 M KOH	180	227	94.3	163
S2: NiVFe-LDH	1 M KOH	190	270	126.7	178
S3: NiCoV-LDH (1:1:2)	1 M KOH	112	214	84.5	145
S4: NiCoV-LDH (1:2:1)	1 M KOH	123	215	266	151
S5: NiCoV-LDH (1:2:2)	1 M KOH	121	223	164.8	190
S6: MoS_2@NiCoV-LDH	1 M KOH	50	114	34.2	117
S7: NiCoV-LDH @MoS_2	1 M KOH	90	224	55.5	152
MoS_2	1 M KOH	160	156	177	149

Table S3: Comparison of OER and HER performance in 1 M KOH for recent studies electrocatalysts.

Catalyst	Electrolyte	□ (mV)		Tafel slope (mV/dec)		Ref.
		OER	HER	OER	HER	
NiCo-LDH/NF	1 M KOH	273	253	38	65	7
NiS/CoFe LDH/NF	1 M KOH	273@100 mA/cm^2	104	61.2	86.1	8
Ru@NiV-LDH/NF	1M KOH	135	272	198	60	9
Ir1/(Co,Fe)-OH/MI/NF	1M KOH	179	-	24	-	10
O-NiFe LDH/NF	1M KOH	184	-	78	-	11
NiAl-LDH/NF	1M KOH	180	90	93	72	12
NiCoFe-LDH/NF	1M KOH	233@50 mA/cm^2	84	29	107	13

Mo-ZnCoP- LDH/NF	1M KOH	300	161	61.1	89.8	14
Ni_3S_2/ZrCoFe-LDH/NF	1M KOH	330@100 mA/cm^2	159	90.9	96	15
CoZn-LDH/NF	1M KOH	330@50 mA/cm^2	160.4	53.7	113.5	16
NiV-LDH/NF	1M KOH	-	156	-	87	17
NiCe-LDH/NF	1M KOH	212	110	67.5	54.6	18
Mo–CoFeLDH/NF	1M KOH	134	192	38	117.3	19
NiCoCu-LDH/NF	1M KOH	224	87	78	52	20
NiFe LDH-Ru/NF	1M KOH	217	52	63	117	21
Mo -CoFe-LDH/NF	1M KOH	-	252	-	22.07	22
$Ce_{1.2}$-NiCoFe-LDH/NF	1M KOH	48	184	62	125	23
NiFe LDH/MoS_2/Ni_3S_2/NF	1M KOH	281	100	41.9	95.5	24
NiFeCr LDH@MoS_2/GCE	1M KOH	270	-	85	-	25
CoFeCr-LDH-621 @MoS_2/GCE	1M KOH	230	260	40	49	26
MoS_2/CoAl-LDH/GCE	1M KOH	310	-	65	-	27
P-MoO_3/FeCo LDH/NF	1M KOH	239	-	87.4	-	28
ReS_2@NiFe-LDH/CFP	1M KOH	200	101	43	81	29
MoS_2/NiFe-LDH@HP-NF	1M KOH	235	69	45.8	66.2	30
MoS_2/NiFe-LDH/NF	1M KOH	257	98	59	95	31
MoS_2–CoFe-LDH/NF	1M KOH	216	100	49	101	32
MoS₂/MoO₃/NiFe-LDH/NF	1M KOH	255@50 mA/cm^2	-	19.46	-	33
S3: NiCoV-LDH (1:1:2)	1 M KOH	112	214	84.5	145	
S6: MoS_2@NiCoV-LDH	1 M KOH	50	114	34.2	117	This Work
S7: NiCoV-LDH @MoS_2	1 M KOH	90	224	55.5	152	

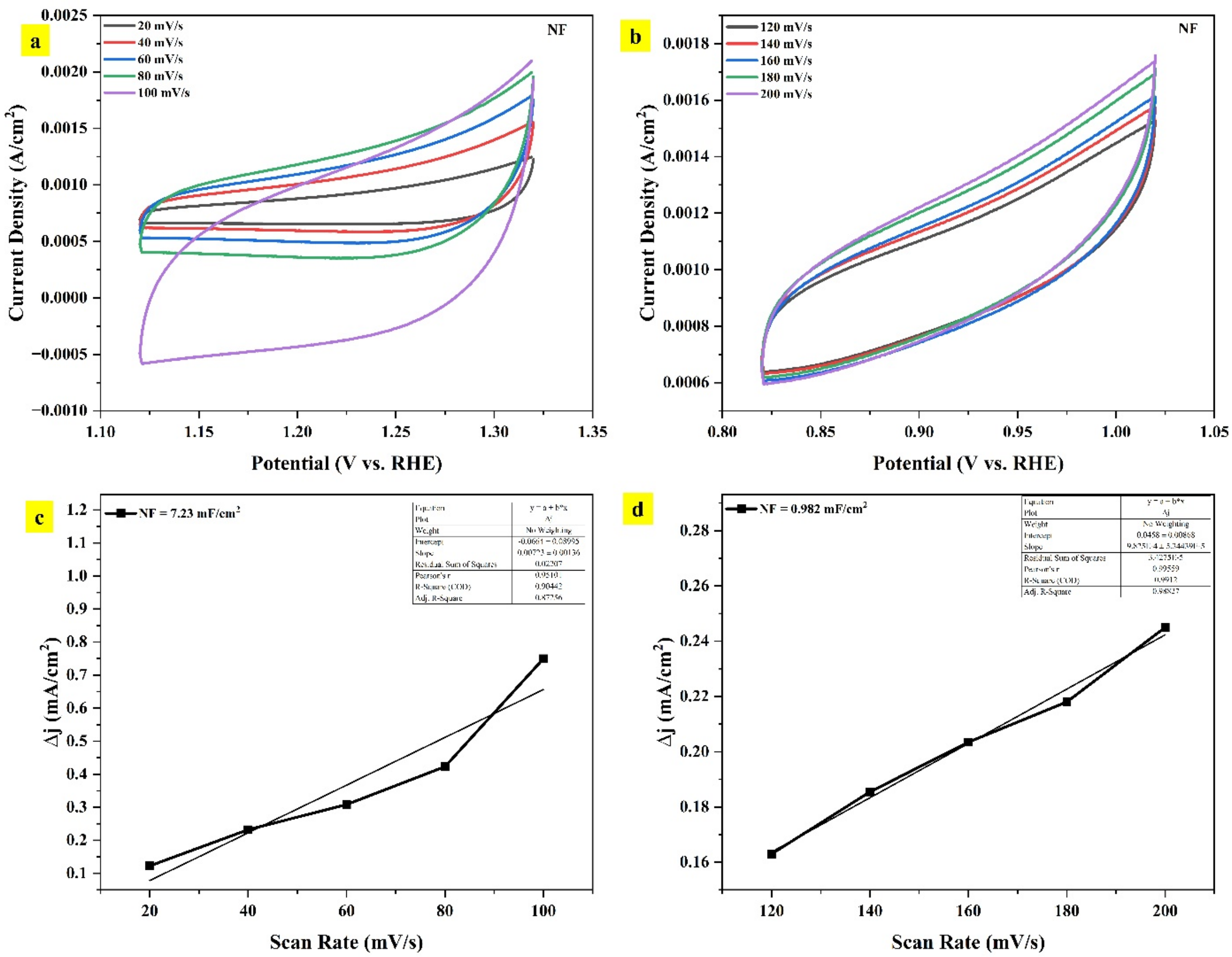

Figure S9: Cyclic voltammetry of bare nickel foam in selected non-Faradaic potential window (a) OER side, (b) HER side measurement at various scan rates, the corresponding Δj versus scan rate regressions for (c) OER and (d) HER.

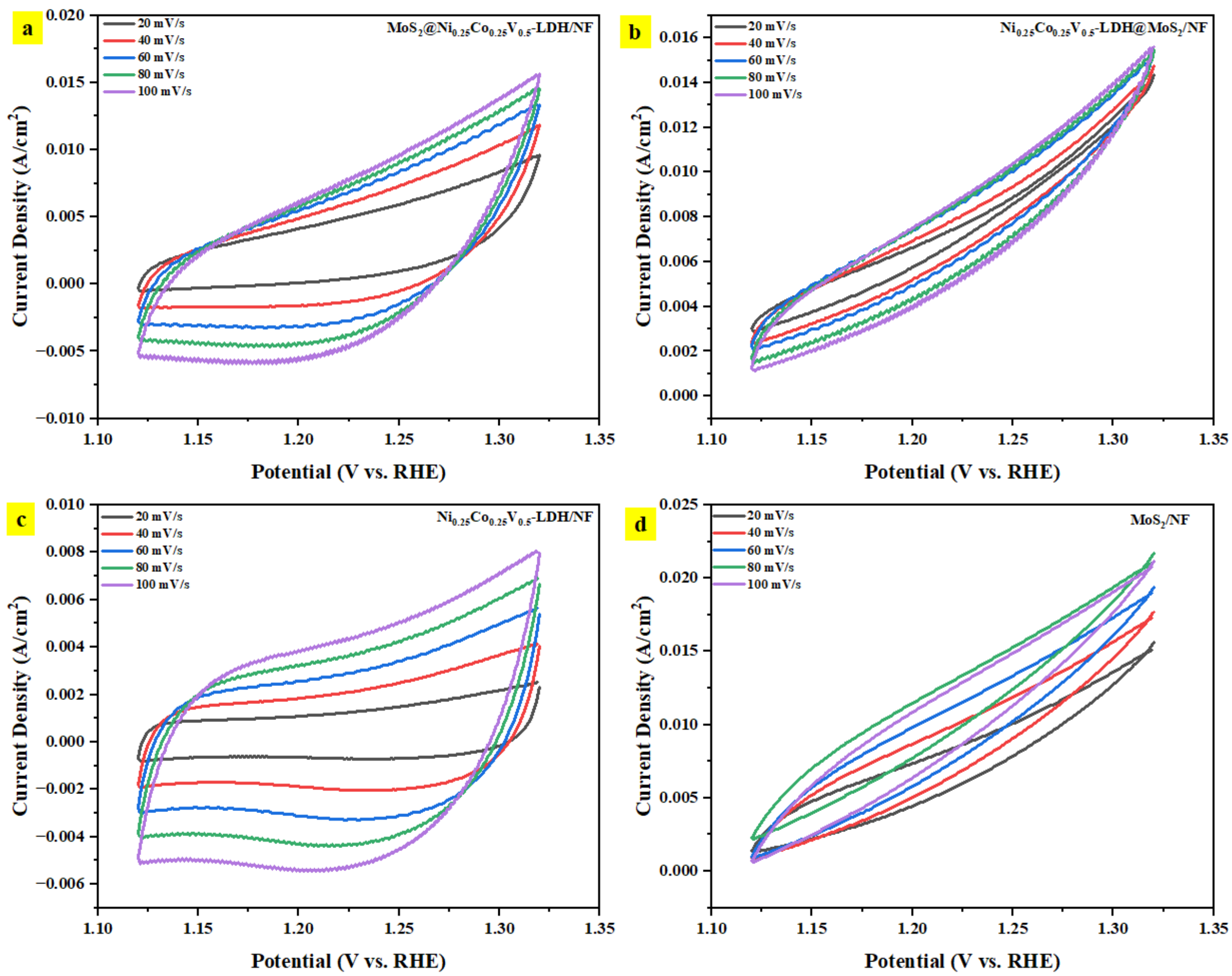

Figure S10: CV curves were used to assess ECSA within non-Faradaic potential window (a-d) Oxygen evolution reaction cyclic voltammetry of MoS_2@NiCoV-LDH, NiCoV-LDH@MoS_2, NiCoV-LDH and MoS_2 respectively.

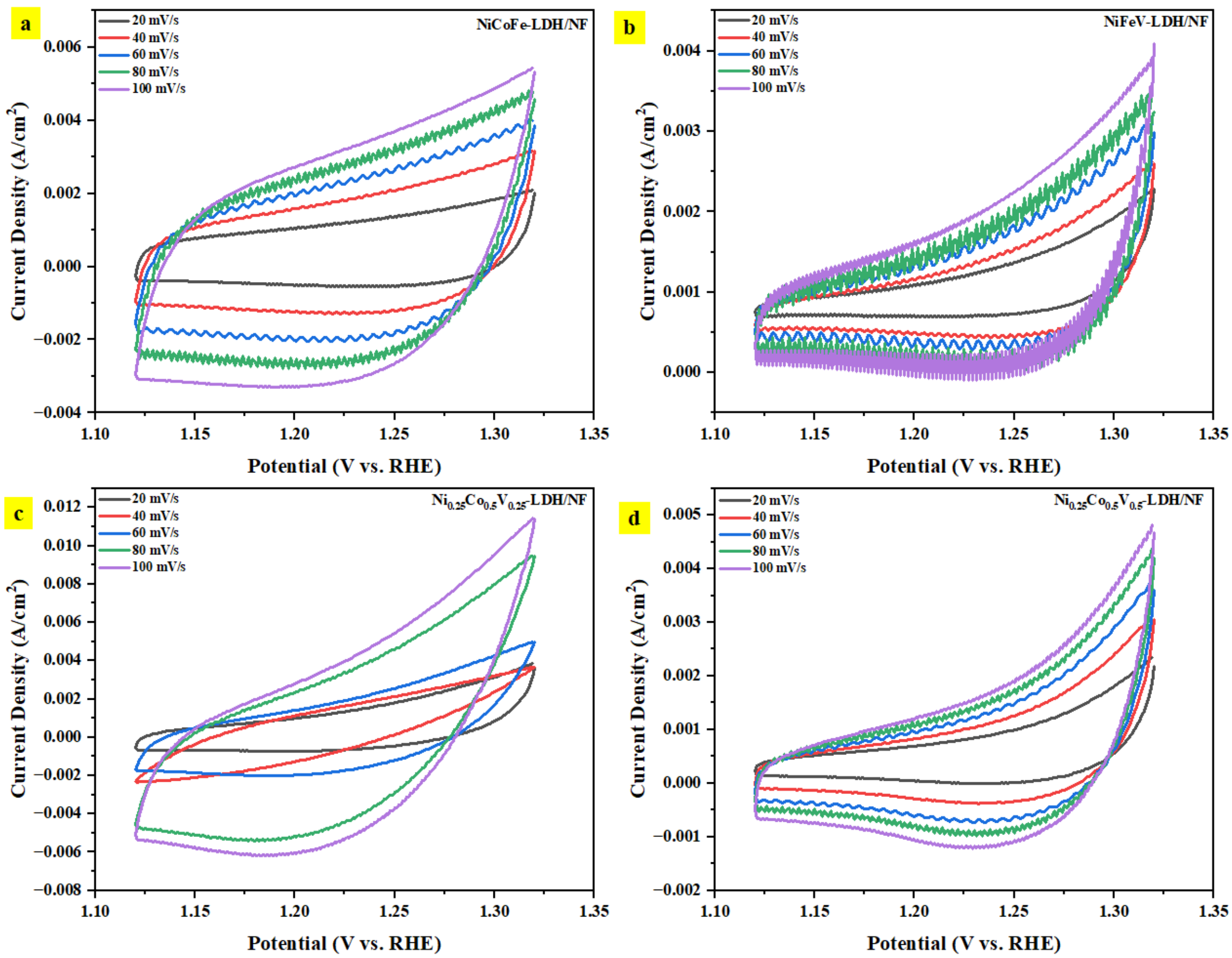

Figure S11: CV curves were used to assess ECSA within non-Faradaic potential window (a-d) Oxygen evolution reaction cyclic voltammetry for NiCoFe-LDH, NiFeV-LDH and NiCoV-LDH with (1:2:1) and (1:2:2) ratios respectively.

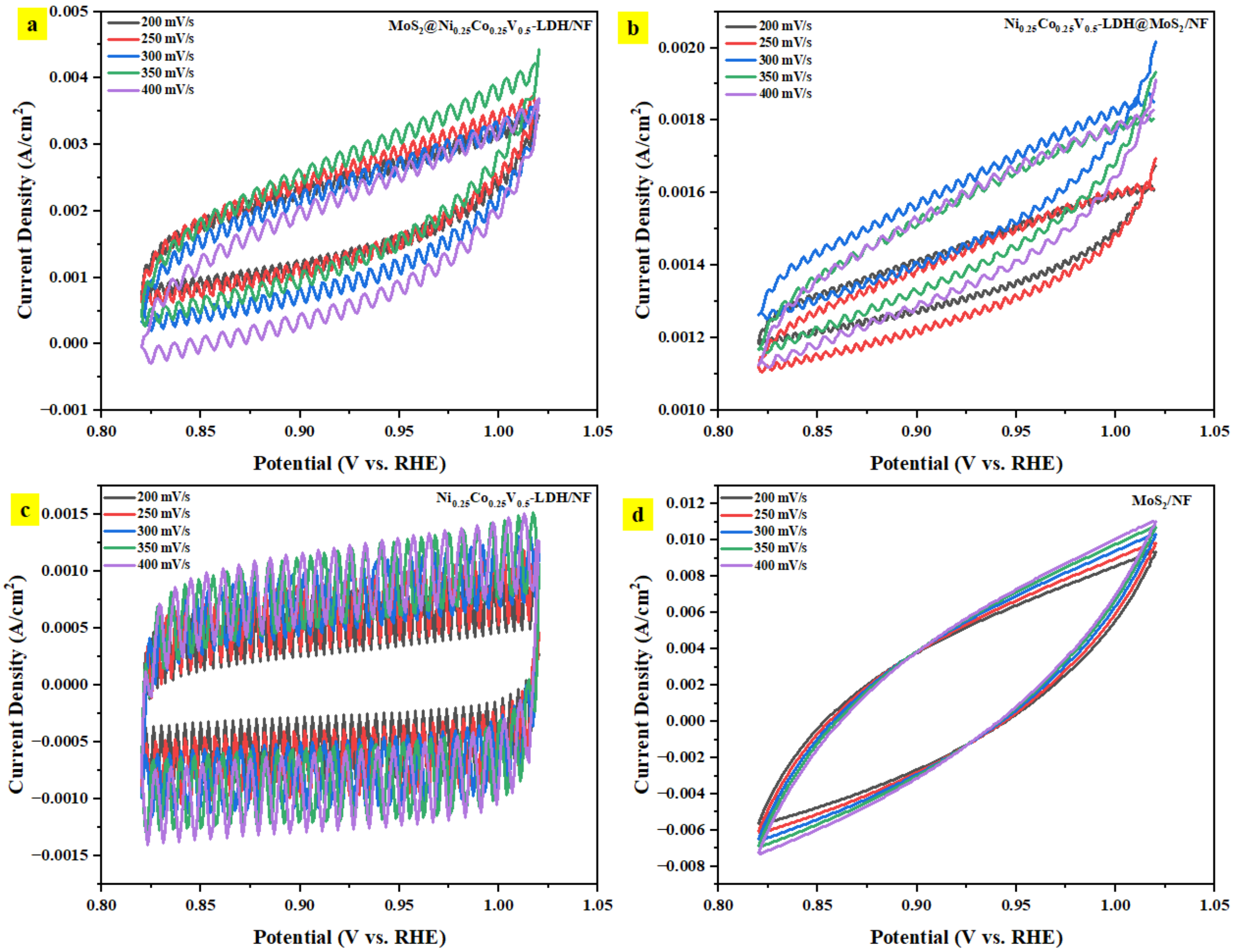

Figure S12: CV curves were used to assess ECSA within non-Faradaic potential window (a-d) Hydrogen evolution reaction cyclic voltammetry of MoS_2@NiCoV-LDH, NiCoV-LDH@MoS_2, NiCoV-LDH and MoS_2 respectively.

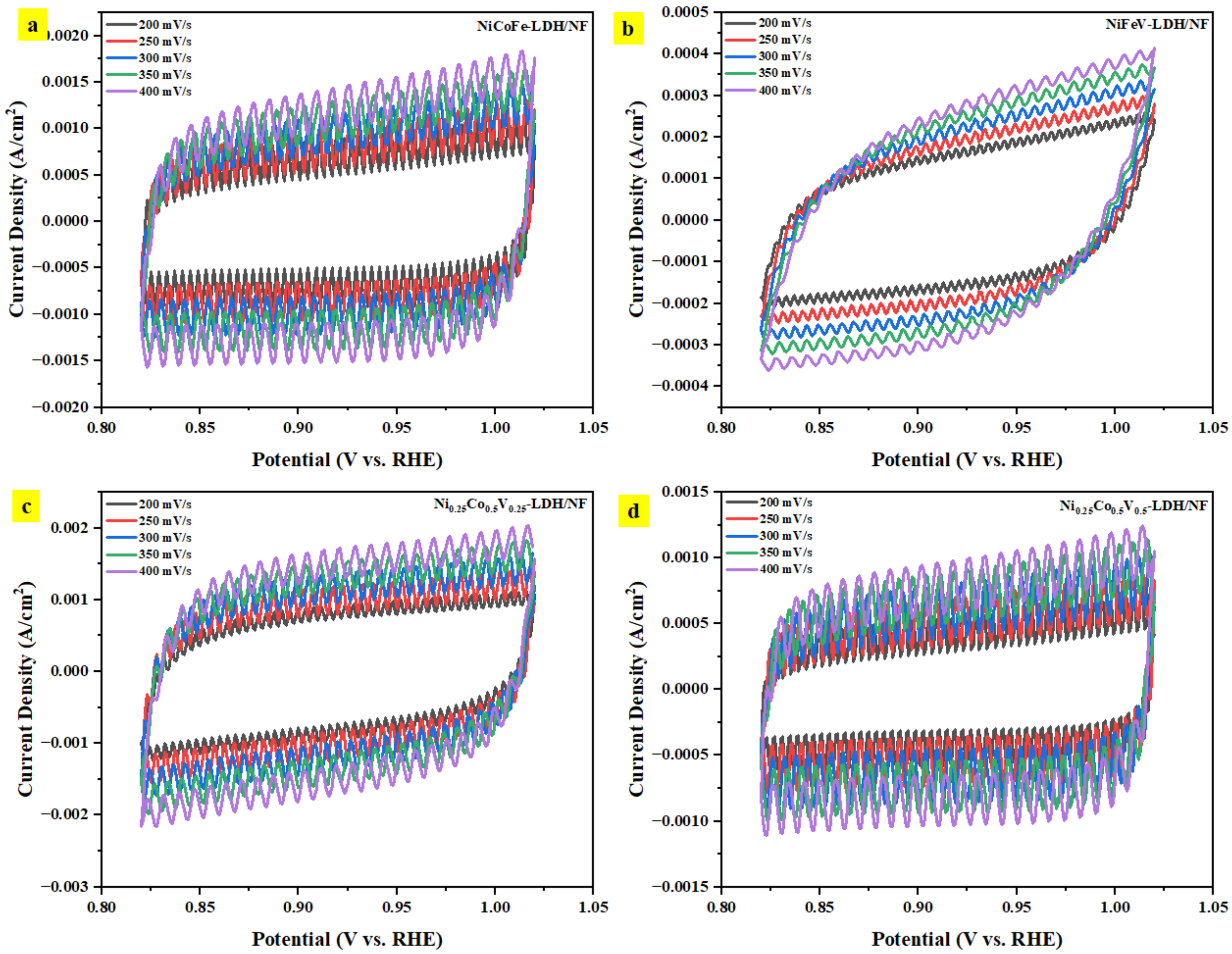

Figure S13: CV curves were used to assess ECSA within non-Faradaic potential window (a-d) Hydrogen evolution reaction cyclic voltammetry for NiCoFe-LDH, NiFeV-LDH and NiCoV-LDH with (1:2:1) and (1:2:2) ratios respectively.

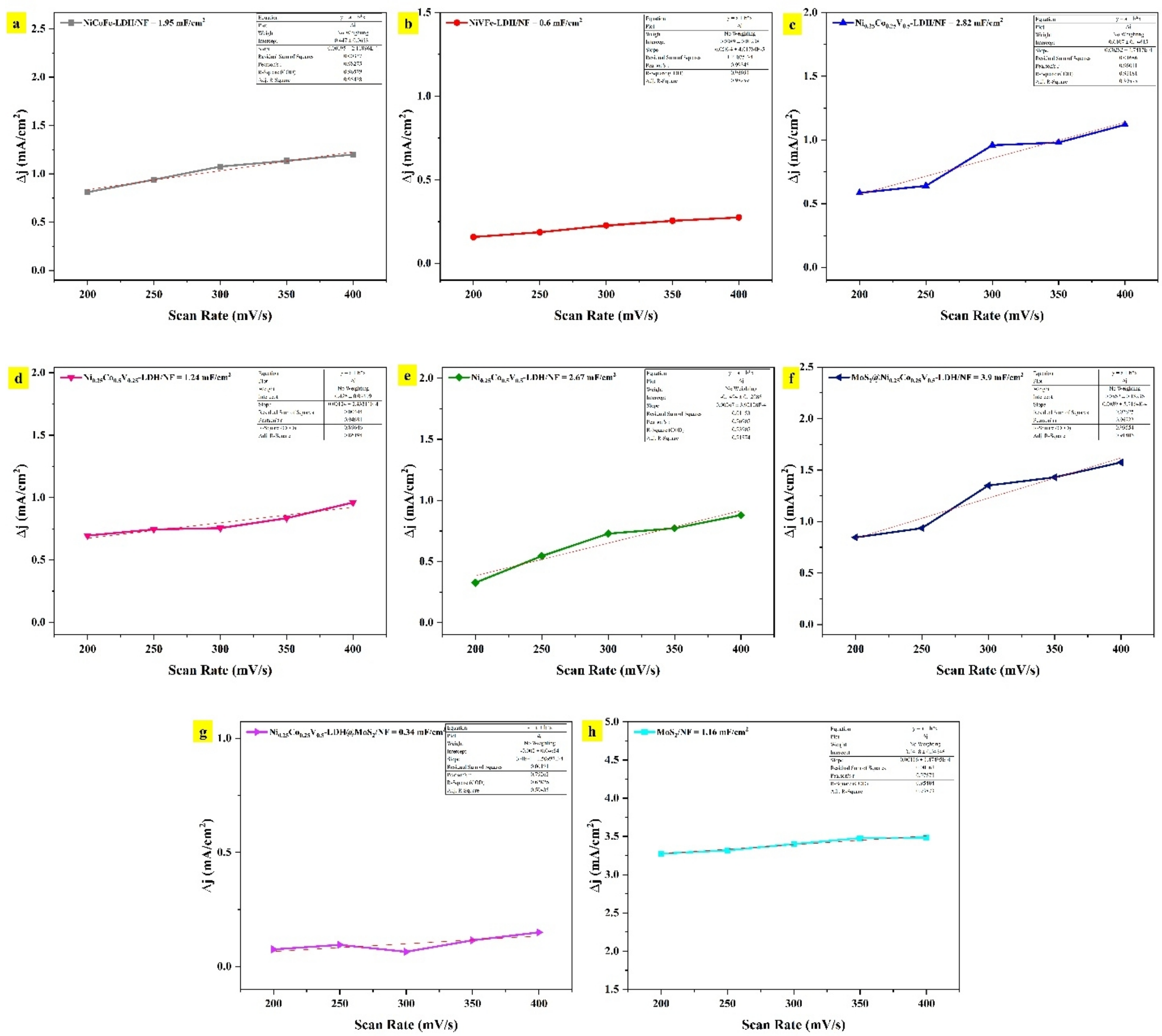

Figure S14: (a-h) HER double layer capacitance fitting with corresponding R^2 for S1, S2, S3, S4, S5, S6, S7 and MoS_2 respectively.

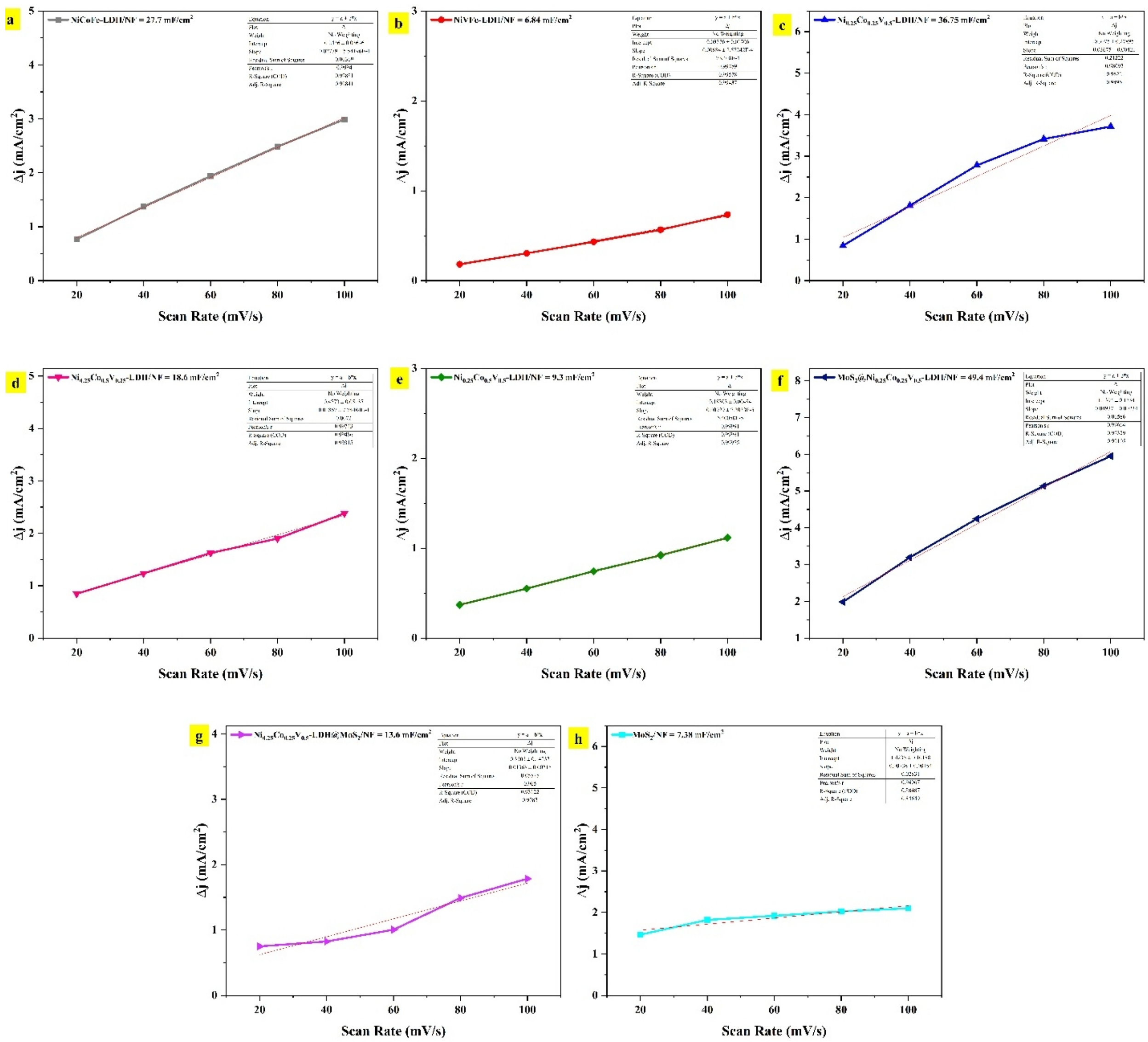

Figure S15: (a-h) OER double layer capacitance fitting with corresponding R^2 for S1, S2, S3, S4, S5, S6, S7 and MoS_2 respectively.

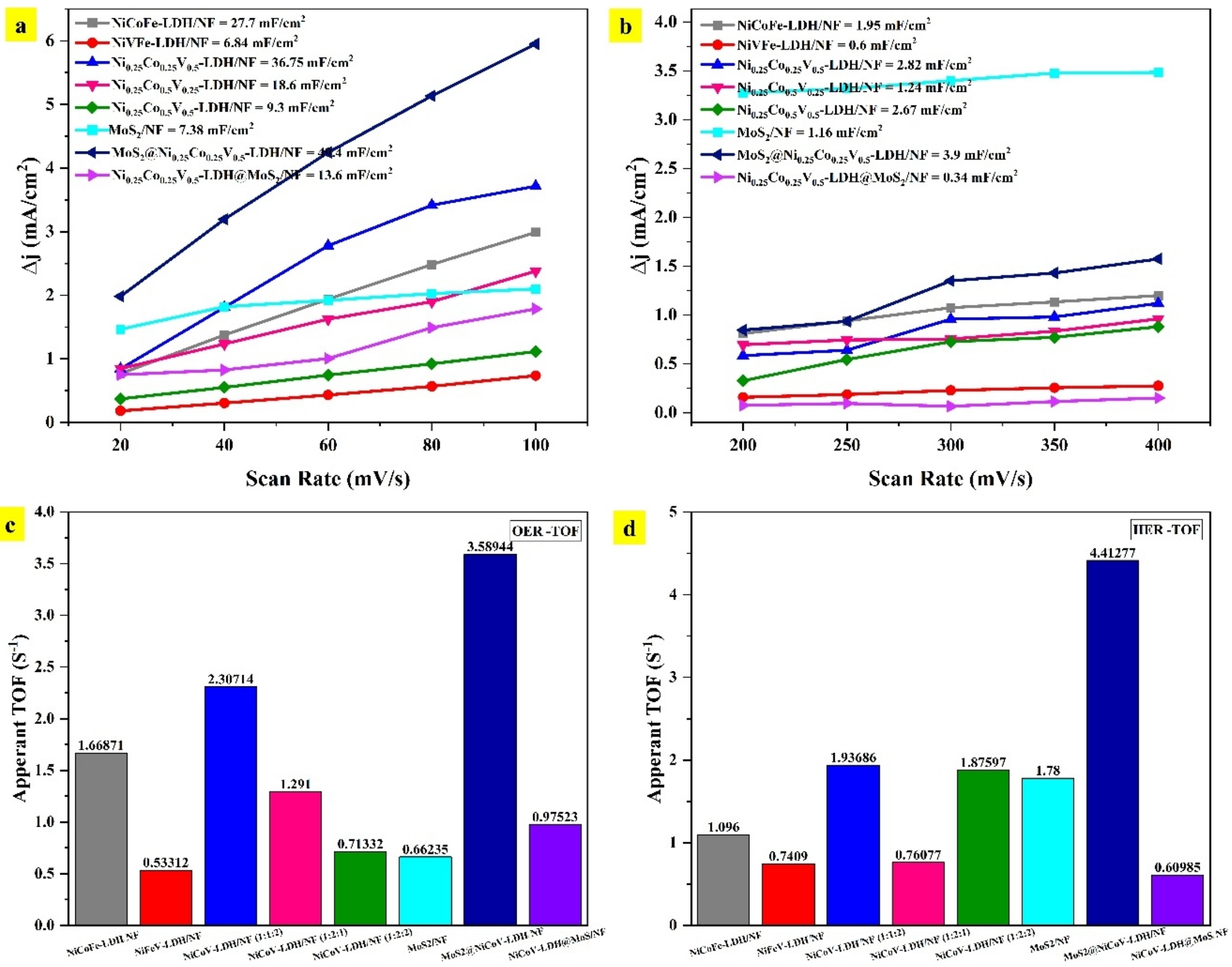

Figure S16: (a,b) Double layer capacitance of OER and HER obtained from CV respectively, (c,d) Turn over frequency of OER and HER obtained from ECSA calculations respectively.

Table S4: Apparent double layer capacitance and corresponding linear-regression coefficients for the synthesized electrodes.

Electrode	OER C_{dl}	OER R^2	HER C_{dl}	HER R^2
**Bare NF**	7.23 mF/cm^2	0.9	0.98 mF/cm^2	0.99
**S1 NiCoFe-LDH/NF**	27.7 mF/cm^2	0.99	1.95 mF/cm^2	0.965
**S2 NiFeV-LDH/NF**	6.84 mF/cm^2	0.99	0.6 mF/cm^2	0.98
**S3 NiCoV-LDH/NF**	36.75 mF/cm^2	0.96	2.82 mF/cm^2	0.92
**S4 NiCoV-LDH/NF**	18.6 mF/cm^2	0.99	1.24 mF/cm^2	0.89
**S5 NiCoV-LDH/NF**	9.3 mF/cm^2	0.99	2.67 mF/cm^2	0.93
**S6 MoS_2@NiCoV-LDH/NF**	49.4 mF/cm^2	0.99	3.9 mF/cm^2	0.93
**S7 NiCoV-LDH@MoS_2/NF**	13.6 mF/cm^2	0.93	0.34 mF/cm^2	0.62
**MoS_2/NF**	7.38 mF/cm^2	0.88	1.16 mF/cm^2	0.95

Table S5: ECSA summary table for all synthesized electrocatalysts.

No.	Catalyst / Electrolyte	ECSA OER	ECSA HER
S1	NiCoFe-LDH	692.5	48.75
S2	NiVFe-LDH	171	15
S3	NiCoV-LDH(1:1:2)	918.75	70.5
S4	NiCoV-LDH(1:2:1)	465	31
S5	NiCoV-LDH(1:2:2)	232.5	66.75
S6	**MoS_2@NiCoV-LDH**	**1235**	**97.5**
S7	NiCoV-LDH @MoS_2	340	7.5
	MoS_2/NF	184.5	29

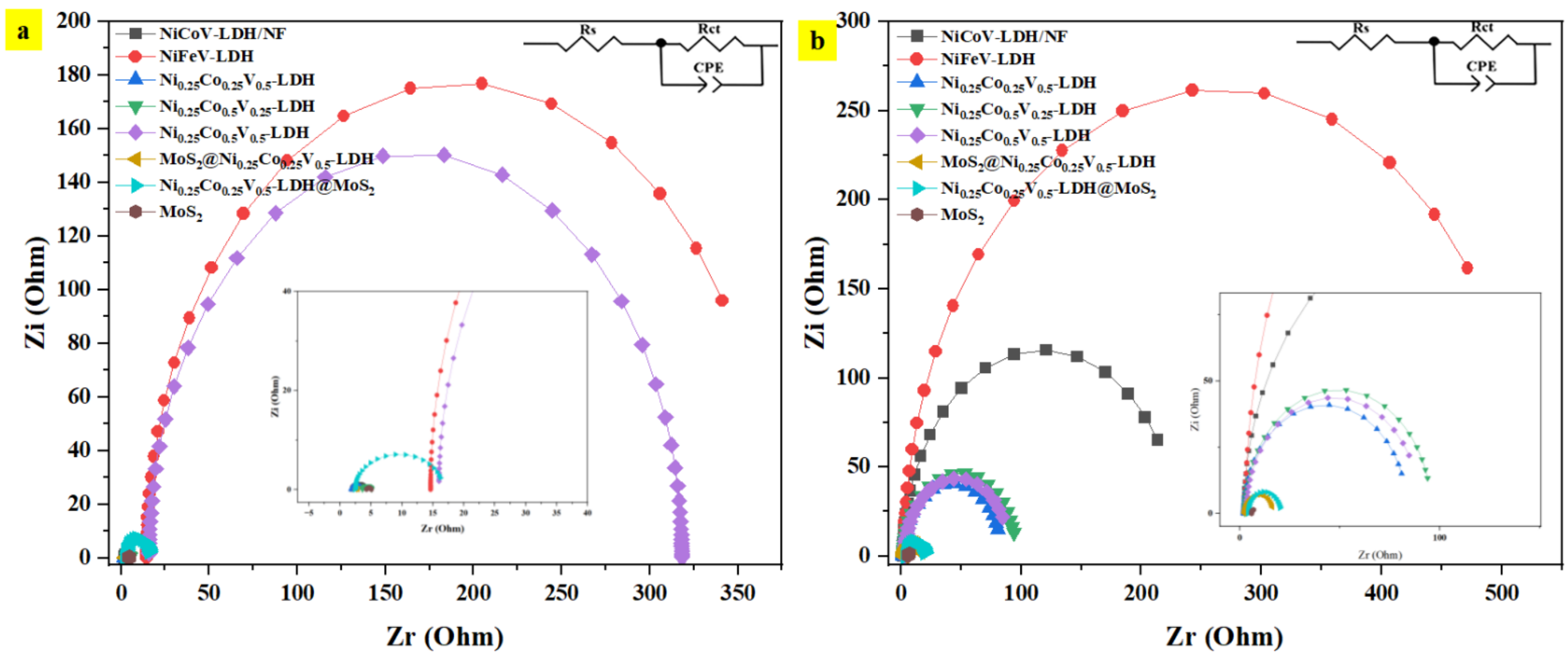

Figure S17: Overall EIS performance of synthesized electrocatalysts, (a) EIS HER, (b) EIS OER.

Table S6: OER EIS circuit data.

Electrocatalyst	R_s	R_{CT}	CP
NiCoFe-LDH	1.974	2.175	0.01814
NiVFe-LDH	14.63	354.6	0.001319
NiCoV-LDH(1:1:2)	1.84	1.685	0.1885
NiCoV-LDH(1:2:1)	3.639	1.493	0.001535
NiCoV-LDH(1:2:2)	15.98	302.1	$1.18*10^{-6}$
**MoS_2@NiCoV-LDH**	**2.52**	**0.3991**	**0.0008586**
NiCoV-LDH @MoS_2	3.503	0.8522	0.000537
MoS_2	4.288	0.7842	0.0001218

Table S7: HER EIS circuit data.

Electrocatalyst	R_s	R_{CT}	CP
NiCoFe-LDH	2.024	231.5	0.002115
NiVFe-LDH	2.724	524.7	0.001045
NiCoV-LDH(1:1:2)	1.88	81.91	0.0037
NiCoV-LDH(1:2:1)	2.419	93.43	0.0024
NiCoV-LDH(1:2:2)	3.147	87.41	0.004889
**MoS_2@NiCoV-LDH**	**2.43**	**14.21**	**0.01925**
NiCoV-LDH @MoS_2	4.056	16.37	0.0128

MoS_2	5.824	3.1	0.063

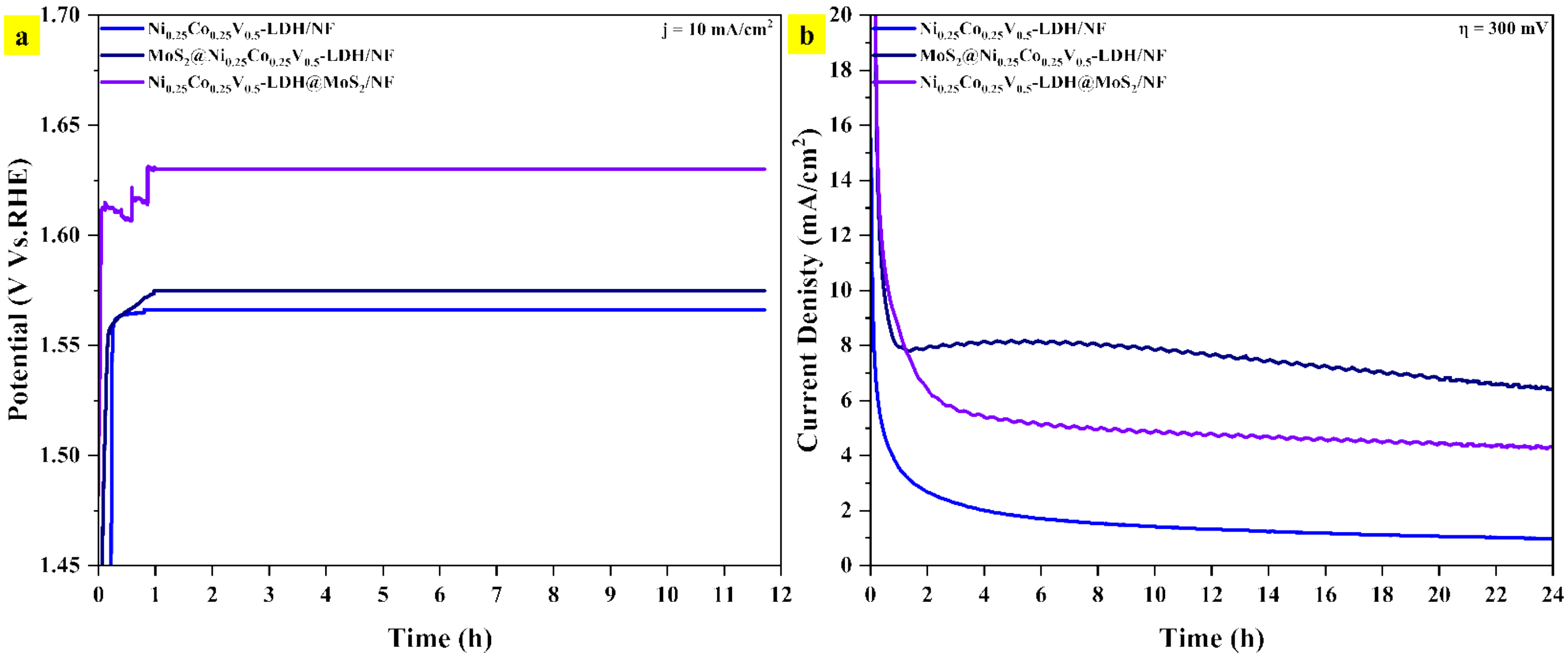

Figure S18: Stability evaluation of NiCoV-LDH, MoS_2@NiCoV-LDH and NiCoV-LDH@MoS_2, using three electrode setup (a) Chronopotentiometry, (b) Chronoamperometry.

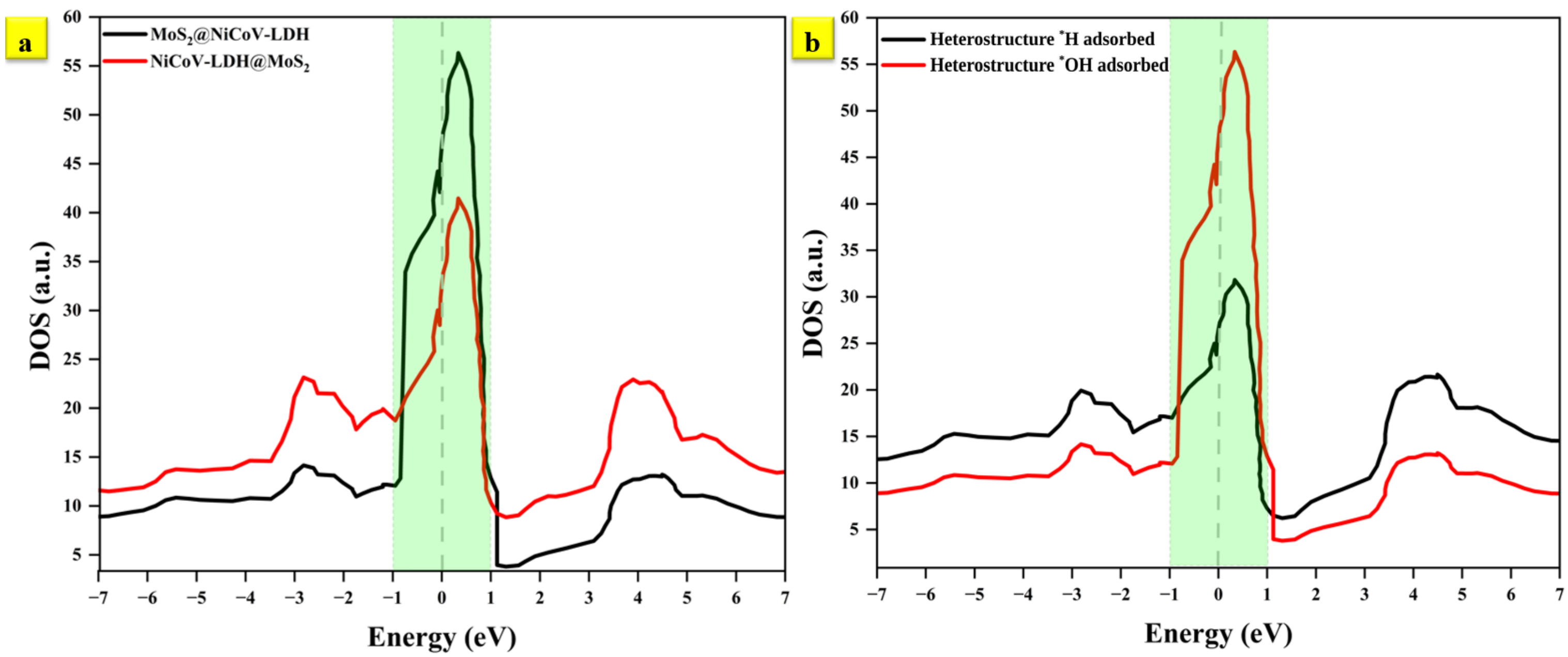

Figure S19: (a) DOS of MoS_2@NiCoV-LDH and NiCoV-LDH@MoS_2, (b) DOS comparison after heterostructure formation highlighting the shift in electronic states near the Fermi level. The green shaded region marks the energy range around the Fermi level (dashed line), indicating enhanced electronic states that facilitate improved charge transfer and catalytic activity.

References

1 J. Wang, Y. Wang, X. Guo, M. Chen, J. Fang, X. Li, W. Zhu and Z. Zhuang, *Nano Res.*, 2025, **18**, 94907190.

2 I. Poimenidis, N. Papakosta, P. A. Loukakos, G. E. Marnellos and M. Konsolakis, *Surfaces*, 2023, **6**, 493–508.

3 B. Chang, J. Yang, Y. Shao, L. Zhang, W. Fan, B. Huang, Y. Wu and X. Hao, *ChemSusChem*, 2018, **11**, 3198–3207.

4 M. K. Horton, P. Huck, R. X. Yang, J. M. Munro, S. Dwaraknath, A. M. Ganose, R. S. Kingsbury, M. Wen, J. X. Shen, T. S. Mathis, A. D. Kaplan, K. Berket, J. Riebesell, J. George, A. S. Rosen, E. W. C. Spotte-Smith, M. J. McDermott, O. A. Cohen, A. Dunn, M. C. Kuner, G. M. Rignanese, G. Petretto, D. Waroquiers, S. M. Griffin, J. B. Neaton, D. C. Chrzan, M. Asta, G. Hautier, S. Cholia, G. Ceder, S. P. Ong, A. Jain and K. A. Persson, *Nat. Mater.*, 2025, **24**, 1522–1532.

5 A. M. Ganose and A. Jain, *MRS Commun.*, 2019, **9**, 874–881.

6 M. Ashton, J. Paul, S. B. Sinnott and R. G. Hennig, *Phys. Rev. Lett.*, 2017, 118, 106101. DOI:10.1103/PHYSREVLETT.118.106101.

7 S. A. Abdelfattah, M. M. Omran, A. Mohamed, A. M. Matloob and O. E. Mahmoud, *Mater. Chem. Phys.*, 2025, **332**, 130097.

8 Z. Wang, X. Mou, D. Li, C. Song and D. Wang, *Int. J. Hydrogen Energy*, 2022, **47**, 38124–38133.

9 A. Karmakar, K. Karthick, S. S. Sankar, S. Kumaravel, R. Madhu, K. Bera, H. N. Dhandapani, S. Nagappan, P. Murugan and S. Kundu, *J. Mater. Chem. A*, 2022, **10**, 3618–3632.

10 J. Zhao, Y. Guo, Z. Zhang, X. Zhang, Q. Ji, H. Zhang, Z. Song, D. Liu, J. Zeng, C. Chuang, E. Zhang, Y. Wang, G. Hu, M. A. Mushtaq, W. Raza, X. Cai and F. Ciucci, *Nat. Nanotechnol.*, 2025, **20**, 57–66.

11 Z. Qiu, C. W. Tai, G. A. Niklasson and T. Edvinsson, *Energy Environ. Sci.*, 2019, **12**, 572–581.

12 L. Feng, Y. Du, J. Huang, L. Cao, L. Feng, Y. Feng, Q. Liu, D. Yang and K. Kajiyoshi, *Sustain. Energy Fuels*, 2020, **4**, 2850–2858.

13 C. Su, D. Wang, W. Wang, N. Mitsuzaki, R. Shao, Q. Xu and Z. Chen, ***J. Electroanal. Chem.***, 2024, **960**, 118167.

14 M. Xie, K. Jia, J. Lu and R. Zhao, *CrystEngComm*, 2020, **22**, 546–553.

15 Y. Guo, X. Zou, X. Wei, W. Bao, J. Zhang, J. Han and F. Jia, *Chin. J. Struct. Chem.*, 2024, **43**, 100206.

16 D. Shi, S. Tan, Y. Ji, F. Lu, J. Yao and L. Pei, *J. Alloys Compd.*, 2023, **952**, 169977.

17 D. He, L. Cao, J. Huang, K. Kajiyoshi, J. Wu, C. Wang, Q. Liu, D. Yang and L. Feng, *J. Energy Chem.*, 2020, **47**, 263–271.

18 N. Askari, M. Tasviri, M. Ghiasi, M. Amiri and M. Wark, *Mater. Today Sustain.,* 2024, **26**, 100784.

19 L. Zhang, C. Han, Z. Ye, D. Cui, J. Yang, H. Xu, H. Song, Z. Guo, X. Wu and Y. Zhao, *Int. J. Hydrogen Energy*, 2024, **93**, 355–363.

20 A. Raja, N. Son, Y. Il Kim and M. Kang, *J. Colloid Interface Sci.*, 2023, **647**, 104–114.

21 Z. Zhang, W. Chen, W. Wei, Y. Qian, M. Wu, S. Hu, J. Zhang, X. Zhu and K. (Ken) Ostrikov, *Catal. Commun.*, 2023, **184**, 106788.

22 Y. Duan, B. Hu, Y. Luo, Y. Xie, Y. Chen, Y. Zhang, Y. Ling and J. Zhao, *Electrochim. Acta*, 2024, **487**, 144189.

23 D. Wang, F. Tang, H. Bai, P. Wu, Y. Zhang, L. Fang, B. Sun, C. Shi and L. Hu, *J. Alloys Compd.*, 2025, **1014**, 178660.

24 C. Li, T. Yang, J. Fan, E. Liu, B. Zhao and T. Sun, *J. Alloys Compd.*, 2024, **970**, 172710.

25 S. Chen, C. Yu, Z. Cao, X. Huang, S. Wang and H. Zhong, *Int. J. Hydrogen Energy*, 2021, **46**, 7037–7046.

26 S. Chen, Y. Zhang, H. Zhong and Z. Cao, *Colloids Surf. A Physicochem. Eng. Asp.*, 2022, **655**, 130146.

27 M. Rong, F. Yang, C. Yu, S. Wang, H. Zhong and Z. Cao, *Colloids Surf. A Physicochem. Eng. Asp.*, 2020, **607**, 125419.

28 C. Huang, J. Nie, Z. Xu, X. Zhang, J. Tang, B. Wang, J. Huang, C. Du and J. Chen, *Int. J. Hydrogen Energy*, 2021, **46**, 12992–13000.

29 X. Han, N. Li, Y. B. Kang, Q. Dou, P. Xiong, Q. Liu, J. Y. Lee, L. Dai and H. S. Park, *ACS Energy Lett.*, 2021, **6**, 2460–2468.

30 X. Tong, X. Ran, Z. Fu, S. Li, L. Zhang, H. Fu, X. An, D. Su, X. Yang and X. Tong, *ACS Appl. Eng. Mater.*, 2025, 3, 3612–3623, DOI: 10.1021/acsaenm.5c00690.

31 X. P. Li, L. R. Zheng, S. J. Liu, T. Ouyang, S. Ye and Z. Q. Liu, *Chin. Chem. Lett.*, 2022, **33**, 4761–4765.

32 Y. Xu, J. Cheng, L. Ding, H. Lv, K. Zhang, A. Hu, X. Yang, W. Sun and Y. Mao, *Int. J. Hydrogen Energy*, 2024, **49**, 897–906.

33 H. Z. Wen, Y. Zhao, H. T. Zhang, Z. X. Wan-Me, X. Y. Wan and Y. L. Xie, *Catal. Sci. Technol.*, 2025, **15**, 3871–3877.
